# A Multi-Parameter Perturbation Solution and Experimental Verification for Bending Problem of Piezoelectric Cantilever Beams

**DOI:** 10.3390/polym11121934

**Published:** 2019-11-24

**Authors:** Zhi-Xin Yang, Xiao-Ting He, Hong-Xia Jing, Jun-Yi Sun

**Affiliations:** 1 School of Civil Engineering, Chongqing University, Chongqing 400045, China; 20141602063@cqu.edu.cn (Z.-X.Y.); jinghongxiajy@163.com (H.-X.J.); sunjunyi@cqu.edu.cn (J.-Y.S.); 2 Key Laboratory of New Technology for Construction of Cities in Mountain Area, Chongqing University, Ministry of Education, Chongqing 400045, China

**Keywords:** multi-parameter perturbation method, piezoelectric polymers, experimental verification, cantilever beam, force–electric coupling characteristics

## Abstract

The existing studies indicate that the application of piezoelectric polymers is becoming more and more extensive, especially in the analysis and design of sensors or actuators, but the problems of piezoelectric structure are usually difficult to solve analytically due to the force–electric coupling characteristics. In this study, the bending problem of a piezoelectric cantilever beam was investigated via theoretical and experimental methods. First, the governing equations of the problem were established and non-dimensionalized. Three piezoelectric parameters were selected as perturbation parameters and the perturbation solution of the equations was finally obtained using a multi-parameter perturbation method. In addition, the relevant experiments of the piezoelectric cantilever beam were carried out, and the experimental results were in good agreement with the theoretical solutions. Based on the experimental results, the effect of piezoelectric properties on the bending deformation of piezoelectric cantilever beams was analyzed and discussed. The results indicated that the multi-parameter perturbation solution obtained in this study is effective and it may serve as a theoretical reference for the design of sensors or actuators made of piezoelectric polymers.

## 1. Introduction

Piezoelectric polymers have been widely used in sensors, actuators, electronic information and intelligent structures because of its great force–electric coupling characteristics [1,2,3,4,5,6]. The piezoelectric polymers usually participate in the work of piezoelectric instruments in the form of piezoelectric sheets which usually are simplified to a piezoelectric cantilever beam [7,8,9].The problems of piezoelectric cantilever beams are usually difficult to be solved analytically due to the existence of the force–electric coupling constitutive relation. It is known that the design of piezoelectric instruments often requires the analytical expression of the problem of piezoelectric cantilever beams as a theoretical reference. Therefore, it is necessary and meaningful to find an efficient analytical method for solving the problem of piezoelectric cantilever beams and giving their analytical solutions.

In the past twenty years, many researchers have studied the problem of piezoelectric cantilever beams and obtained some corresponding solutions. Wang and Chen [10] obtained a general solution of the control equation for the three-dimensional problem of transverse isotropic piezoelectric material by means of a set of new potential functions representing displacement component and potential function, and solved the problem of spatial piezoelectric material under the action of concentrated transverse shear force. Lin et al. [11] derived the analytical expressions of displacement, potential, and stress distribution of piezoelectric beams which were simply supported at both ends under a uniform load. According to the plane stress problem, Mei and Zeng [12] directly derived the equation of state of piezoelectric beams from the piezoelectric physical equation, and on this basis, the exact state equation solution of electromechanical coupling effect of simply supported piezoelectric beams at both ends under a uniform load was given. On the basis of three-dimensional constitutive equations and their simplified equations of elastic piezoelectric materials, Zhu [13] derived the analytic solution to a piezoelectric cantilever beam with concentrated force at the free end in terms of displacements and voltage. For the orthotropic piezoelectric plane problem, Ding et al. [14,15,16] solved a series of piezoelectric beam problems and obtained the corresponding exact solutions with the trial and error method on the basis of the general solution in the case of three distinct eigenvalues, and expressed all displacements, electrical potential, stresses, and electrical displacements by three displacement functions in terms of harmonic polynomials. Yang and Liu [17] investigated the bending of transversely isotropic cantilever beams under an end load, and derived the simplified linear elastic equations of piezoelectric cantilever beams according to the characters of the problem. Pang et al. [18] manufactured a typical Li- and Ta/Sb-modified, alkaline niobate-based, lead-free piezoelectric ceramics by two-step sintering and investigated the sintering condition dependence of dielectric constants and piezoelectric properties. Zhu et al. [19] studied the active vibration control of piezoelectric cantilever beams, where an adaptive feed forward controller (AFC) was utilized to reject the vibration with unknown multiple frequencies. Peng et al. [20] presented time-delayed feedback control to reduce the non-linear resonant vibration of a piezoelectric elastic beam and examined three single-input linear time-delayed feedback control methodologies: displacement, velocity, and acceleration time-delayed feedback. Liu and Yang [21] studied the bending problem of a cantilever beam made of a transversely isotropic piezoelectricity medium under uniformly distributed loads. Shi et al. [22,23] studied the analytical solution of a density functionally gradient piezoelectric cantilever under axial and transverse uniform loads and applied DC voltages and then, solved the force–electric coupling plane strain problem of simply supported beams under a uniform load by the inverse method. Wang et al. [24] dealt with the vibration analysis of a circular plate surface bonded by two piezoelectric layers, based on the Kirchhoff plate model. Recently, Lian et al. [25] studied the problem of a functionally graded piezoelectric cantilever beam under combined loads, but non-dimensionalization was not considered in solving the problem. There is still a lot of research performed in this field, which will not be elaborated here. The summation of results of existing research shows that there are still some unsolved problems. First, non-dimensionalization was not considered in the existing research. We know that piezoelectric materials have not only mechanical properties, but also electrical properties. So, there are both mechanical units and electrical units to be solved, which may lead to computational errors. Second, the existing research basically provides theoretical solutions, but there are a few related experimental verifications. Therefore, the reliability of theoretical solutions cannot be guaranteed. Besides, there has been no unified and effective method for solving the problems of piezoelectric structure.

Parameter perturbation method is a general analysis method for solving approximate solutions of non-linear mechanical problems. It has been successfully applied to various fields of non-linear structural analysis, such as non-linear bending and post-buckling, and has become a powerful tool for solving non-linear problems of structures. Generally speaking, the perturbation method is based on a selected small parameter. In order to solve the problem of parameter selection, Chen and Li [26] put forward the concept of free parameter perturbation method, that is, there is no need to point out the physical meaning of perturbation parameters during perturbation, which provides a new idea for solving the parameter selection problem of parameter perturbation method. Lian et al. [27] solved the Hencky membrane problem without a small-rotation-angle assumption by the single-parameter perturbation method. The successful application of perturbation method depends, to a large extent, on the reasonable choice of small parameters, but the selection of perturbation parameters does not have a set of step-by-step procedures, which can only rely on deep understanding and multiple attempts. To avoid the difficulty in the selection of perturbation parameters, researchers can select multiple parameters, that is, the so-called "multi-parameter perturbation method". For multi-parameter perturbation method, Nowinski and Ismail [28] solved the cylindrical orthotropic circular plate problem under a uniform load by using the two-parameter perturbation method. The application of the multi-parameter perturbation method in beam problem was proposed by Chien [29] in 2002, the classical Euler–Bernoulli equation of bending beams was solved by using load and beam height differences as perturbation parameters. Later, He and Chen [30] simplified the bending moment by using the quasi-linear analysis method, so that the parameter perturbation process was directly aimed at the algebra equation rather than the integral equation, and the two-parameter perturbation solution of the large deflection bending problem of a cantilever beam was obtained, and the integrity of the two-parameter perturbation solution was analyzed. Recently, He et al. [31,32] comprehensively analyzed the large deflection problem of beams with height difference under various boundary conditions, put forward the so-called "two-parameter perturbation method", and successfully applied this method to the solution of bimodular von-Kármán thin plate equation. But so far, the perturbation method of three or more parameters has only a few reports.

In this study, we will derive the theoretical solution of the bending problem of piezoelectric cantilever beams by the multi-parameter perturbation method. The whole paper is organized as follows. In Section 2, the mechanical model of the problem solved here will be established, and the governing equations will be given and dimensionless. In Section 3, the three piezoelectric parameters will be selected as perturbation parameters, and the dimensionless governing equations will be solved by the multi-parameter perturbation method. The solution presented in this paper will be compared with the existing analytical solution from Yang and Liu [17] in Section 4. Next, in Section 5, we will show the related experiments of the piezoelectric cantilever beam, compare the experimental results with the solution presented here, and also discuss the effect of the piezoelectric properties on the deformation of piezoelectric cantilever beams. According to the results mentioned above, some main conclusions will be drawn in Section 6.

## 2. Mechanical Model and Basic Equations

**Figure 1:**
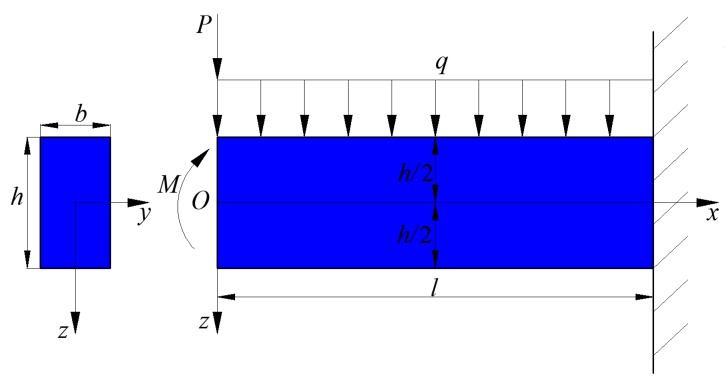
Scheme of a piezoelectric cantilever beam.

In this study, the mechanical model of the transversely isotropic piezoelectric cantilever beam is established by using two-dimensional elastic beam theory and neglecting shear deformation. As shown in Figure 1, an transversely isotropic piezoelectric cantilever beam is fixed at its right end and subjected to a uniformly distributed load q on its upper surface, a concentrated force P and a bending moment M at its left end, in which l, b, and h denote the length, width, and height of the beam, respectively, and O denotes the origin of the coordinates. A rectangular coordinate system is introduced with the upper and lower surfaces of the beam lying in z=−h/2 and z=h/2.

Supposing that the polarization direction is the forward direction of the *z*-axis, let us take a microelement in the piezoelectric cantilever beam, and from the balance of the force, we may obtain, by neglecting the body force

(1) $$\left\{ \begin{array}{l} \frac{\partial \sigma_{x}}{\partial x}+\frac{\partial \tau_{zx}}{\partial z}=0\\ \frac{\partial \tau_{zx}}{\partial x}+\frac{\partial \sigma_{z}}{\partial z}=0 \end{array}\right.,$$

where $\sigma_{x}$, $\sigma_{z}$ and $\tau_{zx}$ are the stress components. The equation of Maxwell electric displacement conservation is

(2) $$\frac{\partial D_{x}}{\partial x}+\frac{\partial D_{z}}{\partial z}=0,$$

where $D_{x}$ and $D_{z}$ are the electric displacement components. The constitutive equations of piezoelectric polymeric materials considered are

(3) $$\left\{ \begin{array}{l} \varepsilon_{x}=s_{11}\sigma_{x}+s_{13}\sigma_{z}+d_{31}E_{z}\\ \varepsilon_{z}=s_{13}\sigma_{x}+s_{33}\sigma_{z}+d_{33}E_{z}\\ \gamma_{zx}=s_{44}\tau_{zx}+d_{15}E_{x}\\ D_{x}=d_{15}\tau_{zx}+\lambda_{11}E_{x}\\ D_{z}=d_{31}\sigma_{x}+d_{33}\sigma_{z}+\lambda_{33}E_{z} \end{array}\right.,$$

where $\varepsilon_{x}$, $\varepsilon_{z}$, and $\gamma_{zx}$ are the strain components; and $E_{x}$ and $E_{z}$ are the electric field intensity components. The geometric equations of the piezoelectric cantilever beam are

(4) $$\varepsilon_{x}=\frac{\partial u}{\partial x},\varepsilon_{z}=\frac{\partial w}{\partial z},\gamma_{zx}=\frac{\partial u}{\partial z}+\frac{\partial w}{\partial x},$$

where u and w are the displacement components. From Equation (4), the strain consistency equation is obtained as follows:

(5) $$\frac{\partial^{2}\varepsilon_{x}}{\partial z^{2}}+\frac{\partial^{2}\varepsilon_{z}}{\partial x^{2}}-\frac{\partial^{2}\gamma_{zx}}{\partial z\partial x}=0.$$

The relationship between electric field intensity and electric potential are

(6) $$E_{x}=-\frac{\partial \phi }{\partial x},E_{z}=-\frac{\partial \phi }{\partial z},$$

where ϕ is the electric potential function. By introducing the Airy stress function U(x,z), the stress components can be expressed as

(7) $$\sigma_{x}=\frac{\partial^{2}U}{\partial z^{2}},\sigma_{z}=\frac{\partial^{2}U}{\partial x^{2}},\tau_{zx}=-\frac{\partial^{2}U}{\partial z\partial x}.$$

The boundary conditions of the problem of the piezoelectric cantilever beam are

(8) $$\left\{ \begin{array}{l} \int_{-h/2}^{h/2}\tau_{zx}dz=\int_{-h/2}^{h/2}\frac{\partial^{2}U}{\partial z\partial x}dz=-\frac{P}{b},\\ \int_{-h/2}^{h/2}\sigma_{x}dz=\int_{-h/2}^{h/2}\frac{\partial^{2}U}{\partial z^{2}}dz=0,\\ \int_{-h/2}^{h/2}z\sigma_{x}dz=\int_{-h/2}^{h/2}z\frac{\partial^{2}U}{\partial z^{2}}dz=\frac{M}{b} \end{array}\right.,\;\mathrm{at}\;x=0,$$

(9) $$\sigma_{z}=\frac{\partial^{2}U}{\partial x^{2}}=0,\tau_{zx}=-\frac{\partial^{2}U}{\partial x\partial z}=0,\;\mathrm{at}\;z=h/2,$$

(10) $$\sigma_{z}=\frac{\partial^{2}U}{\partial x^{2}}=-q,\tau_{zx}=-\frac{\partial^{2}U}{\partial x\partial z}=0,\;\mathrm{at}\;z=-h/2,$$

(11) $$\int_{-h/2}^{h/2}D_{x}dz=0,\;\mathrm{at}\;x=0\;\mathrm{and}\;x=l,$$

(12) $$E_{z}=\frac{\partial \phi }{\partial z}=0,\;\mathrm{at}\;z=\pm h/2,$$

and

(13) $$u=0,w=0,\frac{\partial w}{\partial x}=0,\;\mathrm{at}\;z=0\;\mathrm{and}\;x=l.$$

Substituting Equations (3), (6), and (7) into Equations (2) and (4), we may obtain two equations of the stress function U(x,z) and the potential function ϕ

(14) $$d_{31}\frac{\partial^{3}U}{\partial z^{3}}+d_{33}\frac{\partial^{3}U}{\partial x^{2}\partial z}-d_{15}\frac{\partial^{3}U}{\partial x^{2}\partial z}=\lambda_{33}\frac{\partial^{2}\phi }{\partial z^{2}}+\lambda_{11}\frac{\partial^{2}\phi }{\partial x^{2}}$$

and

(15) $$s_{11}\frac{\partial^{4}U}{\partial z^{4}}+(2s_{13}+s_{44})\frac{\partial^{4}U}{\partial x^{2}\partial z^{2}}+s_{33}\frac{\partial^{4}U}{\partial x^{4}}=d_{31}\frac{\partial^{3}\phi }{\partial z^{3}}+d_{33}\frac{\partial^{3}\phi }{\partial x^{2}\partial z}-d_{15}\frac{\partial^{3}\phi }{\partial x^{2}\partial z}.$$

Equations (14) and (15) are usually called governing equations. Let us introduce the following dimensionless quantities:

(16) $$\begin{array}{l} X=\frac{x}{h},Z=\frac{z}{h},S_{13}=\frac{s_{13}}{s_{11}},S_{33}=\frac{s_{33}}{s_{11}},S_{44}=\frac{s_{44}}{s_{11}},{\bar{d}}_{31}=\frac{d_{31}}{\sqrt{s_{11}\lambda_{11}}},{\bar{d}}_{33}=\frac{d_{33}}{\sqrt{s_{11}\lambda_{11}}},{\bar{d}}_{15}=\frac{d_{15}}{\sqrt{s_{11}\lambda_{11}}},\\ \Phi =\frac{\phi \sqrt{s_{11}\lambda_{11}}}{h},{\bar{\lambda }}_{33}=\frac{\lambda_{33}}{\lambda_{11}},\bar{P}=\frac{P}{h^{2}}s_{11},\bar{b}=\frac{b}{h},\bar{M}=\frac{M}{h^{3}}s_{11},\bar{q}=qs_{11},\bar{u}=\frac{u}{h},\bar{w}=\frac{w}{h},\bar{U}=\frac{Us_{11}}{h^{2}} \end{array}$$

From Equation (16), Equations (14) and (15) can be transformed into

(17) $${\bar{d}}_{31}\frac{\partial^{3}\bar{U}}{\partial Z^{3}}+{\bar{d}}_{33}\frac{\partial^{3}\bar{U}}{\partial X^{2}\partial Z}-{\bar{d}}_{15}\frac{\partial^{3}\bar{U}}{\partial X^{2}\partial Z}={\bar{\lambda }}_{33}\frac{\partial^{2}\Phi }{\partial Z^{2}}+\frac{\partial^{2}\Phi }{\partial X^{2}}$$

and

(18) $$\frac{\partial^{4}\bar{U}}{\partial Z^{4}}+(2S_{13}+S_{44})\frac{\partial^{4}\bar{U}}{\partial X^{2}\partial Z^{2}}+S_{33}\frac{\partial^{4}\bar{U}}{\partial X^{4}}={\bar{d}}_{31}\frac{\partial^{3}\Phi }{\partial Z^{3}}+{\bar{d}}_{33}\frac{\partial^{3}\Phi }{\partial X^{2}\partial Z}-{\bar{d}}_{15}\frac{\partial^{3}\Phi }{\partial X^{2}\partial Z}.$$

The boundary conditions can be transformed into

(19) $$\int_{-1/2}^{1/2}\frac{\partial^{2}\bar{U}}{\partial Z\partial X}dZ=-\frac{\bar{P}}{\bar{b}},\int_{-1/2}^{1/2}\frac{\partial^{2}\bar{U}}{\partial Z^{2}}dZ=0,\int_{-1/2}^{1/2}Z\frac{\partial^{2}\bar{U}}{\partial Z^{2}}dZ=\frac{\bar{M}}{\bar{b}},\;\mathrm{at}\;X=0,$$

(20) $$\frac{\partial^{2}\bar{U}}{\partial X^{2}}=-\frac{\partial^{2}\bar{U}}{\partial X\partial Z}=0,\;\mathrm{at}\;Z=1/2,$$

(21) $$\frac{\partial^{2}\bar{U}}{\partial X^{2}}=-\bar{q},-\frac{\partial^{2}\bar{U}}{\partial X\partial Z}=0,\;\mathrm{at}\;Z=-1/2,$$

(22) $$\int_{-1/2}^{1/2}\left(-{\bar{d}}_{15}\frac{\partial^{2}\bar{U}}{\partial Z\partial X}-\frac{\partial \Phi }{\partial X}\right)dZ=0,\;\mathrm{at}\;X=0\;\mathrm{and}\;X=l/h,$$

(23) $$\frac{\partial \Phi }{\partial Z}=0,\;\mathrm{at}\;Z=\pm 1/2,$$

and

(24) $$\bar{u}=0,\bar{w}=0,\frac{\partial \bar{w}}{\partial X}=0,\;\mathrm{at}\;Z=0\;\mathrm{and}\;X=l/h.$$

## 3. Multi-parameter Perturbation Solution

Equations (17) and (18) are two partial differential equations which are usually difficult to solve analytically. Here, we use the multi-parameter perturbation method to solve them. The piezoelectric coefficients are usually very small [33], thus, they can be selected as perturbation parameters to meet the requirement of convergence in perturbation expansions. From the point of view of the perturbation idea, if the cantilever beam without piezoelectric properties is regarded as an unperturbed system, the piezoelectric cantilever beam can be looked upon as a perturbed system. Selecting ${\bar{d}}_{31}$, ${\bar{d}}_{33}$ and ${\bar{d}}_{15}$ as the perturbation parameters, the Φ and $\bar{U}$ can be expanded as

(25) $$\begin{array}{l} \Phi =\Phi_{0}^{0}+\Phi_{1}^{Ι}{\bar{d}}_{31}+\Phi_{2}^{Ι}{\bar{d}}_{33}+\Phi_{3}^{Ι}{\bar{d}}_{15}+\Phi_{1}^{\mathrm{ΙΙ}}{\left({\bar{d}}_{31}\right)}^{2}+\Phi_{2}^{\mathrm{ΙΙ}}{\left({\bar{d}}_{33}\right)}^{2}\\ +\Phi_{3}^{\mathrm{ΙΙ}}{\left({\bar{d}}_{15}\right)}^{2}+\Phi_{4}^{\mathrm{ΙΙ}}{\bar{d}}_{31}{\bar{d}}_{33}+\Phi_{5}^{\mathrm{ΙΙ}}{\bar{d}}_{31}{\bar{d}}_{15}+\Phi_{6}^{\mathrm{ΙΙ}}{\bar{d}}_{33}{\bar{d}}_{15} \end{array}$$

and

(26) $$\begin{array}{l} \bar{U}={\bar{U}}_{0}^{0}+{\bar{U}}_{1}^{Ι}{\bar{d}}_{31}+{\bar{U}}_{2}^{Ι}{\bar{d}}_{33}+{\bar{U}}_{3}^{Ι}{\bar{d}}_{15}+{\bar{U}}_{1}^{\mathrm{ΙΙ}}{\left({\bar{d}}_{31}\right)}^{2}+{\bar{U}}_{2}^{\mathrm{ΙΙ}}{\left({\bar{d}}_{33}\right)}^{2}\\ +{\bar{U}}_{3}^{\mathrm{ΙΙ}}{\left({\bar{d}}_{15}\right)}^{2}+{\bar{U}}_{4}^{\mathrm{ΙΙ}}{\bar{d}}_{31}{\bar{d}}_{33}+{\bar{U}}_{5}^{\mathrm{ΙΙ}}{\bar{d}}_{31}{\bar{d}}_{15}+{\bar{U}}_{6}^{\mathrm{ΙΙ}}{\bar{d}}_{33}{\bar{d}}_{15} \end{array},$$

where $\Phi_{0}^{0}$ and ${\bar{U}}_{0}^{0}$, $\Phi_{i}^{Ι}$ and ${\bar{U}}_{i}^{Ι}$ (i=1,2,3), and $\Phi_{i}^{\mathrm{ΙΙ}}$ and ${\bar{U}}_{i}^{\mathrm{ΙΙ}}$ (i=1,2,…,5,6) are unknown functions of X and Z.

First, we solve the zero-order perturbation equations. Substituting Equations (25) and (26) into Equations (17) and (18) and comparing the coefficients of ${\left({\bar{d}}_{31}\right)}^{0}$, ${\left({\bar{d}}_{33}\right)}^{0}$ and ${\left({\bar{d}}_{15}\right)}^{0}$, we may obtain the zero-order perturbation equations

(27) $$\left\{ \begin{array}{l} {\bar{\lambda }}_{33}\frac{\partial^{2}\Phi_{0}^{0}}{\partial Z^{2}}+\frac{\partial^{2}\Phi_{0}^{0}}{\partial X^{2}}=0\\ \frac{\partial^{4}{\bar{U}}_{0}^{0}}{\partial Z^{4}}+(2S_{13}+S_{44})\frac{\partial^{4}{\bar{U}}_{0}^{0}}{\partial X^{2}\partial Z^{2}}+S_{33}\frac{\partial^{4}{\bar{U}}_{0}^{0}}{\partial X^{4}}=0 \end{array}\right..$$

The corresponding boundary conditions are

(28) $$\int_{-1/2}^{1/2}(-\frac{\partial^{2}{\bar{U}}_{0}^{0}}{\partial Z\partial X})dZ=-\frac{\bar{P}}{\bar{b}},\int_{-1/2}^{1/2}\frac{\partial^{2}{\bar{U}}_{0}^{0}}{\partial Z^{2}}dZ=0,\int_{-1/2}^{1/2}Z\frac{\partial^{2}{\bar{U}}_{0}^{0}}{\partial Z^{2}}dZ=\frac{\bar{M}}{\bar{b}},\;\mathrm{at}\;X=0,$$

(29) $$\frac{\partial^{2}{\bar{U}}_{0}^{0}}{\partial X^{2}}=-\frac{\partial^{2}{\bar{U}}_{0}^{0}}{\partial X\partial Z}=0,\;\mathrm{at}\;Z=1/2,$$

(30) $$\frac{\partial^{2}{\bar{U}}_{0}^{0}}{\partial X^{2}}=-\bar{q},-\frac{\partial^{2}{\bar{U}}_{0}^{0}}{\partial X\partial Z}=0,\;\mathrm{at}\;Z=-1/2,$$

(31) $$\int_{-1/2}^{1/2}\left(-\frac{\partial \Phi_{0}^{0}}{\partial X}\right)dZ=0,\;\mathrm{at}\;X=0\;\mathrm{and}\;X=l/h$$

and

(32) $$\frac{\partial \Phi_{0}^{0}}{\partial Z}=0,\;\mathrm{at}\;Z=\pm 1/2.$$

Suppose,

(33) $$\left\{ \begin{array}{l} \Phi_{0}^{0}=X^{2}g_{1}^{0}(Z)+Xg_{2}^{0}(Z)+g_{3}^{0}(Z)\\ {\bar{U}}_{0}^{0}=\frac{X^{2}}{2}f_{1}^{0}(Z)+Xf_{2}^{0}(Z)+f_{3}^{0}(Z) \end{array}\right.,$$

where $g_{i}^{0}(Z)$ and $f_{i}^{0}(Z)$ (i=1,2,3) are unknown functions of Z which can be determined by Equations (27) and (33), please see Appendix A.

Next, let us solve the first-order perturbation equations. Comparing the coefficients of ${\left({\bar{d}}_{31}\right)}^{1}$,${\left({\bar{d}}_{33}\right)}^{1}$ and ${\left({\bar{d}}_{15}\right)}^{1}$, we may obtain the first-order perturbation equations as follows.

For term ${\left({\bar{d}}_{31}\right)}^{1}$:

(34) $$\left\{ \begin{array}{l} \frac{\partial^{3}{\bar{U}}_{0}^{0}}{\partial Z^{3}}-{\bar{\lambda }}_{33}\frac{\partial^{2}\Phi_{1}^{Ι}}{\partial Z^{2}}-\frac{\partial^{2}\Phi_{1}^{Ι}}{\partial X^{2}}=0\\ \frac{\partial^{4}{\bar{U}}_{1}^{Ι}}{\partial Z^{4}}+(2S_{13}+S_{44})\frac{\partial^{4}{\bar{U}}_{1}^{Ι}}{\partial X^{2}\partial Z^{2}}+S_{33}\frac{\partial^{4}{\bar{U}}_{1}^{Ι}}{\partial X^{4}}-\frac{\partial^{3}\Phi_{0}^{0}}{\partial Z^{3}}=0 \end{array}\right.,$$

for term ${\left({\bar{d}}_{33}\right)}^{1}$:

(35) $$\left\{ \begin{array}{l} \frac{\partial^{3}{\bar{U}}_{0}^{0}}{\partial X^{2}\partial Z}={\bar{\lambda }}_{33}\frac{\partial^{2}\Phi_{2}^{Ι}}{\partial Z^{2}}+\frac{\partial^{2}\Phi_{2}^{Ι}}{\partial X^{2}}\\ \frac{\partial^{4}{\bar{U}}_{2}^{Ι}}{\partial Z^{4}}+(2S_{13}+S_{44})\frac{\partial^{4}{\bar{U}}_{2}^{Ι}}{\partial X^{2}\partial Z^{2}}+S_{33}\frac{\partial^{4}{\bar{U}}_{2}^{Ι}}{\partial X^{4}}-\frac{\partial^{3}\Phi_{0}^{0}}{\partial X^{2}\partial Z}=0 \end{array}\right.,$$

and for term ${\left({\bar{d}}_{15}\right)}^{1}$:

(36) $$\left\{ \begin{array}{l} -\frac{\partial^{3}{\bar{U}}_{0}^{0}}{\partial X^{2}\partial Z}={\bar{\lambda }}_{33}\frac{\partial^{2}\Phi_{3}^{Ι}}{\partial Z^{2}}+\frac{\partial^{2}\Phi_{3}^{Ι}}{\partial X^{2}}\\ \frac{\partial^{4}{\bar{U}}_{3}^{Ι}}{\partial Z^{4}}+(2S_{13}+S_{44})\frac{\partial^{4}{\bar{U}}_{3}^{Ι}}{\partial X^{2}\partial Z^{2}}+S_{33}\frac{\partial^{4}{\bar{U}}_{3}^{Ι}}{\partial X^{4}}+\frac{\partial^{3}\Phi_{0}^{0}}{\partial X^{2}\partial Z}=0 \end{array}\right..$$

The corresponding boundary conditions are

(37) $$\left\{ \begin{array}{l} \int_{-1/2}^{1/2}-\frac{\partial^{2}{\bar{U}}_{1}^{Ι}}{\partial Z\partial X}dZ=0,\\ \int_{-1/2}^{1/2}\frac{\partial^{2}{\bar{U}}_{2}^{Ι}}{\partial Z^{2}}dZ=0,\\ \int_{-1/2}^{1/2}Z\frac{\partial^{2}{\bar{U}}_{3}^{Ι}}{\partial Z^{2}}dZ=0 \end{array}\right.,\;\mathrm{at}\;X=0,$$

(38) $$\frac{\partial^{2}{\bar{U}}_{i}^{Ι}}{\partial X^{2}}=-\frac{\partial^{2}{\bar{U}}_{i}^{Ι}}{\partial X\partial Z}=0(i=1,2,3),\;\mathrm{at}\;Z=1/2,$$

(39) $$\frac{\partial^{2}{\bar{U}}_{i}^{Ι}}{\partial X^{2}}=0,-\frac{\partial^{2}{\bar{U}}_{i}^{Ι}}{\partial X\partial Z}=0(i=1,2,3),\;\mathrm{at}\;Z=-1/2,$$

(40) $$\left\{ \begin{array}{l} \int_{-1/2}^{1/2}\left(-\frac{\partial \Phi_{1}^{Ι}}{\partial X}\right)dZ=0\\ \int_{-1/2}^{1/2}\left(-\frac{\partial \Phi_{2}^{Ι}}{\partial X}\right)dZ=0\\ \int_{-1/2}^{1/2}\left(-\frac{\partial^{2}{\bar{U}}_{0}^{0}}{\partial Z\partial X}-\frac{\partial \Phi_{3}^{Ι}}{\partial X}\right)dZ=0 \end{array}\right.,\;\mathrm{at}\;X=0\;\mathrm{and}\;X=l/h,$$

and

(41) $$\frac{\partial \Phi_{i}^{Ι}}{\partial Z}=0(i=1,2,3),\;\mathrm{at}\;Z=\pm 1/2.$$

Similarly, suppose

(42) $$\left\{ \begin{array}{l} \Phi_{i}^{Ι}=X^{2}g_{3i-2}^{Ι}(Z)+Xg_{3i-1}^{Ι}(Z)+g_{3i}^{Ι}(Z)\\ {\bar{U}}_{i}^{Ι}=\frac{X^{2}}{2}f_{3i-2}^{Ι}(Z)+Xf_{3i-1}^{Ι}(Z)+f_{3i}^{Ι}(Z) \end{array}\right.(i=1,2,3),$$

where $g_{i}^{Ι}(Z)$ and $f_{i}^{Ι}(Z)$ (i=1,2,3,…,9) are unknown functions of Z which can be determined by Equations (34)–(36) and (42), please see Appendix A.

Then, we solve the second-order perturbation equations. Comparing the coefficients of ${\left(d_{31}\right)}^{2}$, ${\left(d_{33}\right)}^{2}$, ${\left(d_{15}\right)}^{2}$, $d_{31}d_{33}$, $d_{31}d_{15}$ and $d_{33}d_{15}$, we may obtain the two-order perturbation equations as follows.

For term ${\left(d_{31}\right)}^{2}$:

(43) $$\left\{ \begin{array}{l} \frac{\partial^{3}{\bar{U}}_{1}^{Ι}}{\partial Z^{3}}={\bar{\lambda }}_{33}\frac{\partial^{2}\Phi_{1}^{\mathrm{ΙΙ}}}{\partial Z^{2}}+\frac{\partial^{2}\Phi_{1}^{\mathrm{ΙΙ}}}{\partial X^{2}}\\ \frac{\partial^{4}{\bar{U}}_{1}^{\mathrm{ΙΙ}}}{\partial Z^{4}}+(2S_{13}+S_{44})\frac{\partial^{4}{\bar{U}}_{1}^{\mathrm{ΙΙ}}}{\partial X^{2}\partial Z^{2}}+S_{33}\frac{\partial^{4}{\bar{U}}_{1}^{\mathrm{ΙΙ}}}{\partial X^{4}}=\frac{\partial^{3}\Phi_{1}^{Ι}}{\partial Z^{3}} \end{array}\right.,$$

for term ${\left(d_{33}\right)}^{2}$:

(44) $$\left\{ \begin{array}{l} \frac{\partial^{3}{\bar{U}}_{2}^{Ι}}{\partial X^{2}\partial Z}={\bar{\lambda }}_{33}\frac{\partial^{2}\Phi_{2}^{\mathrm{ΙΙ}}}{\partial Z^{2}}+\frac{\partial^{2}\Phi_{2}^{\mathrm{ΙΙ}}}{\partial X^{2}}\\ \frac{\partial^{4}{\bar{U}}_{2}^{\mathrm{ΙΙ}}}{\partial Z^{4}}+(2S_{13}+S_{44})\frac{\partial^{4}{\bar{U}}_{2}^{\mathrm{ΙΙ}}}{\partial X^{2}\partial Z^{2}}+S_{33}\frac{\partial^{4}{\bar{U}}_{2}^{\mathrm{ΙΙ}}}{\partial X^{4}}=\frac{\partial^{3}\Phi_{2}^{Ι}}{\partial X^{2}\partial Z} \end{array}\right.,$$

for term ${\left(d_{15}\right)}^{2}$:

(45) $$\left\{ \begin{array}{l} -\frac{\partial^{3}{\bar{U}}_{3}^{Ι}}{\partial X^{2}\partial Z}={\bar{\lambda }}_{33}\frac{\partial^{2}\Phi_{3}^{\mathrm{ΙΙ}}}{\partial Z^{2}}+\frac{\partial^{2}\Phi_{3}^{\mathrm{ΙΙ}}}{\partial X^{2}}\\ \frac{\partial^{4}{\bar{U}}_{3}^{\mathrm{ΙΙ}}}{\partial Z^{4}}+(2S_{13}+S_{44})\frac{\partial^{4}{\bar{U}}_{3}^{\mathrm{ΙΙ}}}{\partial X^{2}\partial Z^{2}}+S_{33}\frac{\partial^{4}{\bar{U}}_{3}^{\mathrm{ΙΙ}}}{\partial X^{4}}=-\frac{\partial^{3}\Phi_{3}^{Ι}}{\partial X^{2}\partial Z} \end{array}\right.,$$

for term $d_{31}d_{33}$:

(46) $$\left\{ \begin{array}{l} \frac{\partial^{3}{\bar{U}}_{2}^{Ι}}{\partial Z^{3}}+\frac{\partial^{3}{\bar{U}}_{1}^{Ι}}{\partial X^{2}\partial Z}={\bar{\lambda }}_{33}\frac{\partial^{2}\Phi_{4}^{\mathrm{ΙΙ}}}{\partial Z^{2}}+\frac{\partial^{2}\Phi_{4}^{\mathrm{ΙΙ}}}{\partial X^{2}}\\ \frac{\partial^{4}{\bar{U}}_{4}^{\mathrm{ΙΙ}}}{\partial Z^{4}}+(2S_{13}+S_{44})\frac{\partial^{4}{\bar{U}}_{4}^{\mathrm{ΙΙ}}}{\partial X^{2}\partial Z^{2}}+S_{33}\frac{\partial^{4}{\bar{U}}_{4}^{\mathrm{ΙΙ}}}{\partial X^{4}}=\frac{\partial^{3}\Phi_{2}^{Ι}}{\partial Z^{3}}+\frac{\partial^{3}\Phi_{1}^{Ι}}{\partial X^{2}\partial Z} \end{array}\right.,$$

for term $d_{31}d_{15}$:

(47) $$\left\{ \begin{array}{l} \frac{\partial^{3}{\bar{U}}_{3}^{Ι}}{\partial Z^{3}}-\frac{\partial^{3}{\bar{U}}_{1}^{Ι}}{\partial X^{2}\partial Z}={\bar{\lambda }}_{33}\frac{\partial^{2}\Phi_{5}^{\mathrm{ΙΙ}}}{\partial Z^{2}}+\frac{\partial^{2}\Phi_{5}^{\mathrm{ΙΙ}}}{\partial X^{2}}\\ \frac{\partial^{4}{\bar{U}}_{5}^{\mathrm{ΙΙ}}}{\partial Z^{4}}+(2S_{13}+S_{44})\frac{\partial^{4}{\bar{U}}_{5}^{\mathrm{ΙΙ}}}{\partial X^{2}\partial Z^{2}}+S_{33}\frac{\partial^{4}{\bar{U}}_{5}^{\mathrm{ΙΙ}}}{\partial X^{4}}=\frac{\partial^{3}\Phi_{3}^{Ι}}{\partial Z^{3}}-\frac{\partial^{3}\Phi_{1}^{Ι}}{\partial X^{2}\partial Z} \end{array}\right.,$$

and for term $d_{33}d_{15}$:

(48) $$\left\{ \begin{array}{l} \frac{\partial^{3}{\bar{U}}_{3}^{Ι}}{\partial X^{2}\partial Z}-\frac{\partial^{3}{\bar{U}}_{2}^{Ι}}{\partial X^{2}\partial Z}={\bar{\lambda }}_{33}\frac{\partial^{2}\Phi_{6}^{\mathrm{ΙΙ}}}{\partial Z^{2}}+\frac{\partial^{2}\Phi_{6}^{\mathrm{ΙΙ}}}{\partial X^{2}}\\ \frac{\partial^{4}{\bar{U}}_{6}^{\mathrm{ΙΙ}}}{\partial Z^{4}}+(2S_{13}+S_{44})\frac{\partial^{4}{\bar{U}}_{6}^{\mathrm{ΙΙ}}}{\partial X^{2}\partial Z^{2}}+S_{33}\frac{\partial^{4}{\bar{U}}_{6}^{\mathrm{ΙΙ}}}{\partial X^{4}}=\frac{\partial^{3}\Phi_{3}^{Ι}}{\partial X^{2}\partial Z}-\frac{\partial^{3}\Phi_{2}^{Ι}}{\partial X^{2}\partial Z} \end{array}\right..$$

The corresponding boundary conditions are

(49) $$\left\{ \begin{array}{l} \int_{-1/2}^{1/2}\frac{\partial^{2}{\bar{U}}_{1}^{\mathrm{ΙΙ}}}{\partial Z\partial X}dZ=0,\\ \int_{-1/2}^{1/2}\frac{\partial^{2}{\bar{U}}_{2}^{\mathrm{ΙΙ}}}{\partial Z^{2}}dZ=0,\\ \int_{-1/2}^{1/2}Z\frac{\partial^{2}{\bar{U}}_{3}^{\mathrm{ΙΙ}}}{\partial Z^{2}}dZ=0 \end{array}\right.,\;\mathrm{at}\;X=0,$$

(50) $$\frac{\partial^{2}{\bar{U}}_{i}^{\mathrm{ΙΙ}}}{\partial X^{2}}=-\frac{\partial^{2}{\bar{U}}_{i}^{\mathrm{ΙΙ}}}{\partial X\partial Z}=0(i=1,2,3,4,5,6),\;\mathrm{at}\;Z=1/2,$$

(51) $$\frac{\partial^{2}{\bar{U}}_{i}^{\mathrm{ΙΙ}}}{\partial X^{2}}=0,-\frac{\partial^{2}{\bar{U}}_{i}^{\mathrm{ΙΙ}}}{\partial X\partial Z}=0(i=1,2,3,4,5,6),\;\mathrm{at}\;Z=-1/2,$$

(52) $$\left\{ \begin{array}{l} \int_{-1/2}^{1/2}\frac{\partial \Phi_{1}^{\mathrm{ΙΙ}}}{\partial X}dZ=0,\;\int_{-1/2}^{1/2}\frac{\partial \Phi_{2}^{\mathrm{ΙΙ}}}{\partial X}dZ=0\\ \int_{-1/2}^{1/2}\left(\frac{\partial^{2}{\bar{U}}_{3}^{Ι}}{\partial X\partial Z}+\frac{\partial \Phi_{3}^{\mathrm{ΙΙ}}}{\partial X}\right)dZ=0,\;\int_{-1/2}^{1/2}\frac{\partial \Phi_{4}^{\mathrm{ΙΙ}}}{\partial X}dZ=0\\ \int_{-1/2}^{1/2}\left(\frac{\partial^{2}{\bar{U}}_{1}^{Ι}}{\partial X\partial Z}+\frac{\partial \Phi_{5}^{\mathrm{ΙΙ}}}{\partial X}\right)dZ=0,\;\int_{-1/2}^{1/2}\left(\frac{\partial^{2}{\bar{U}}_{2}^{Ι}}{\partial X\partial Z}+\frac{\partial \Phi_{6}^{\mathrm{ΙΙ}}}{\partial X}\right)dZ=0 \end{array}\right.,\;\mathrm{at}\;X=0\;\mathrm{and}\;X=l/h,$$

and

(53) $$\frac{\partial \Phi_{i}^{\mathrm{ΙΙ}}}{\partial Z}=0,\;\mathrm{at}\;Z=\pm 1/2.$$

Suppose,

(54) $$\left\{ \begin{array}{l} \Phi_{i}^{\mathrm{ΙΙ}}=X^{2}g_{3i-2}^{\mathrm{ΙΙ}}(Z)+Xg_{3i-1}^{\mathrm{ΙΙ}}(Z)+g_{3i}^{\mathrm{ΙΙ}}(Z)\\ {\bar{U}}_{i}^{\mathrm{ΙΙ}}=\frac{X^{2}}{2}f_{3i-2}^{\mathrm{ΙΙ}}(Z)+Xf_{3i-1}^{\mathrm{ΙΙ}}(Z)+f_{3i}^{\mathrm{ΙΙ}}(Z) \end{array}\right.(i=1,2,3,4,5,6),$$

where $g_{i}^{\mathrm{ΙΙ}}(Z)$ and $f_{i}^{\mathrm{ΙΙ}}(Z)$ (i=1,2,3,…,18) are unknown functions of Z which can be determined by Equations (43)–(48) and (54), please see Appendix A.

Thus, we can obtain

(55) $$\begin{array}{l} \Phi =B_{6}^{0}+{\bar{d}}_{31}[X^{2}(-\frac{3}{{\bar{\lambda }}_{33}}\bar{q}Z^{2}+\frac{1}{4{\bar{\lambda }}_{33}}\bar{q})+X(-\frac{6}{\bar{b}{\bar{\lambda }}_{33}}\bar{P}Z^{2}+\frac{1}{2\bar{b}{\bar{\lambda }}_{33}}\bar{P})+\frac{1}{2{\left({\bar{\lambda }}_{33}\right)}^{2}}\bar{q}Z^{4}-\frac{1}{4{\left({\bar{\lambda }}_{33}\right)}^{2}}\bar{q}Z^{2}\\ +\frac{\left(2S_{13}+S_{44}\right)}{2{\bar{\lambda }}_{33}}\bar{q}Z^{4}+\frac{6}{\bar{b}{\bar{\lambda }}_{33}}\bar{M}Z^{2}-\frac{3(2S_{13}+S_{44})}{20{\bar{\lambda }}_{33}}\bar{q}Z^{2}+B_{6}^{Ι}]+{\bar{d}}_{33}[-\frac{1}{2{\bar{\lambda }}_{33}}\bar{q}Z^{4}+\frac{3}{4{\bar{\lambda }}_{33}}\bar{q}Z^{2}\\ -\frac{1}{2{\bar{\lambda }}_{33}}\bar{q}Z+B_{12}^{Ι}]+{\bar{d}}_{15}[-\frac{1}{2}\bar{q}X^{2}-\frac{\bar{P}}{\bar{b}}X+\frac{1}{2{\bar{\lambda }}_{33}}\bar{q}Z^{2}+\frac{1}{2{\bar{\lambda }}_{33}}\bar{q}Z^{4}-\frac{3}{4{\bar{\lambda }}_{33}}\bar{q}Z^{2}+B_{18}^{Ι}]\\ +{\left({\bar{d}}_{31}\right)}^{2}B_{6}^{\mathrm{ΙΙ}}+{\left({\bar{d}}_{33}\right)}^{2}B_{12}^{\mathrm{ΙΙ}}+{\left({\bar{d}}_{15}\right)}^{2}B_{18}^{\mathrm{ΙΙ}}+{\bar{d}}_{31}{\bar{d}}_{33}B_{24}^{\mathrm{ΙΙ}}+{\bar{d}}_{31}{\bar{d}}_{15}B_{30}^{\mathrm{ΙΙ}}+{\bar{d}}_{33}{\bar{d}}_{15}B_{36}^{\mathrm{ΙΙ}} \end{array}$$

and

(56) $$\begin{array}{l} \bar{U}=\frac{X^{2}}{2}(-2\bar{q}Z^{3}+\frac{3}{2}\bar{q}Z-\frac{\bar{q}}{2})+X(-\frac{2}{\bar{b}}\bar{P}Z^{3}+\frac{3}{2\bar{b}}\bar{P}Z+C_{8}^{0})+\frac{\left(2S_{13}+S_{44}\right)}{10}\bar{q}Z^{5}+\frac{2}{\bar{b}}\bar{M}Z^{3}\\ -\frac{\left(2S_{13}+S_{44}\right)}{20}\bar{q}Z^{3}+C_{11}^{0}Z+C_{12}^{0}+{\bar{d}}_{31}(XC_{8}^{Ι}+C_{11}^{Ι}Z+C_{12}^{Ι})+{\bar{d}}_{33}(XC_{20}^{Ι}+C_{23}^{Ι}Z+C_{24}^{Ι})\\ +{\bar{d}}_{15}(XC_{32}^{Ι}+C_{35}^{Ι}Z+C_{36}^{Ι})+{\left({\bar{d}}_{31}\right)}^{2}[XC_{8}^{\mathrm{ΙΙ}}+\frac{1}{10{\left({\bar{\lambda }}_{33}\right)}^{2}}\bar{q}Z^{5}+\frac{\left(2S_{13}+S_{44}\right)}{10{\bar{\lambda }}_{33}}\bar{q}Z^{5}-\frac{1}{20{\left({\bar{\lambda }}_{33}\right)}^{2}}\bar{q}Z^{3}\\ -\frac{\left(2S_{13}+S_{44}\right)}{20{\bar{\lambda }}_{33}}\bar{q}Z^{3}+C_{11}^{\mathrm{ΙΙ}}Z+C_{12}^{\mathrm{ΙΙ}}]+{\left({\bar{d}}_{33}\right)}^{2}(XC_{20}^{\mathrm{ΙΙ}}+C_{23}^{\mathrm{ΙΙ}}Z+C_{24}^{\mathrm{ΙΙ}})+{\left({\bar{d}}_{15}\right)}^{2}(XC_{32}^{\mathrm{ΙΙ}}+C_{35}^{\mathrm{ΙΙ}}Z+C_{36}^{\mathrm{ΙΙ}})\\ +{\bar{d}}_{31}{\bar{d}}_{33}(XC_{44}^{\mathrm{ΙΙ}}-\frac{1}{5{\bar{\lambda }}_{33}}\bar{q}Z^{5}+\frac{1}{10{\bar{\lambda }}_{33}}\bar{q}Z^{3}+C_{47}^{\mathrm{ΙΙ}}Z+C_{48}^{\mathrm{ΙΙ}})+{\bar{d}}_{31}{\bar{d}}_{15}(XC_{56}^{\mathrm{ΙΙ}}+\frac{1}{5{\bar{\lambda }}_{33}}\bar{q}Z^{5}-\frac{1}{10{\bar{\lambda }}_{33}}\bar{q}Z^{3}\\ +C_{59}^{\mathrm{ΙΙ}}Z+C_{60}^{\mathrm{ΙΙ}})+{\bar{d}}_{33}{\bar{d}}_{15}(XC_{68}^{\mathrm{ΙΙ}}+C_{71}^{\mathrm{ΙΙ}}Z+C_{72}^{\mathrm{ΙΙ}}) \end{array}.$$

Finally, from Equations (55) and (56), we can obtain the expression of displacement components, stress components, and electric displacement components. The detailed derivation is shown in Appendix B. Thus, the bending problem of a piezoelectric cantilever beam under combined loads is solved. It can be seen from the derivation above that the piezoelectric effect is not shown in the zero-order perturbation solution, that is, the zero-order perturbation solution is the solution of the cantilever beam without piezoelectric properties which is regarded as the unperturbed system. The piezoelectric properties are only shown in the first-order and second-order perturbation solutions. In other words, the mechanical meaning of the first-order and second-order solutions is the influence of piezoelectric properties on the deformation of piezoelectric cantilever beams. This phenomenon is consistent with the basic idea of perturbation method.

## 4. Comparison of the Solution Presented Here and the Existing Solution

The theoretical solution for a piezoelectric cantilever beam under combined loads is given in this paper by a new method which is usually called the multi-parameter perturbation method. The validity of the theoretical solution should further be verified. For this purpose, we compare the solution presented here with the solution given in reference [17].

Before the comparison, we need to make a degradation of the solution presented here. In reference [17], only the concentrated force is considered. In this paper, however, the concentrated force, bending moment, and uniformly distributed load are all considered. Thus, for the convenience of comparison, we let the bending moment and uniformly distributed load equal to zero, that is, let

(57) $$q=0{,}_{}^{}M=0.$$

Substituting Equation (57) into Equations (A44) and (A45), the displacement components can be transformed into

(58) $$\begin{array}{l} w=(\frac{d_{33}d_{31}}{\lambda_{33}}-s_{13})\frac{6P}{bh^{3}}xz^{2}-(\frac{d_{31}d_{31}}{\lambda_{33}}-s_{11})\frac{2P}{bh^{3}}x^{3}\\ +(\frac{d_{31}d_{31}}{\lambda_{33}}-s_{11})\frac{6P}{bh^{3}}l^{2}x-(\frac{d_{31}d_{31}}{\lambda_{33}}-s_{11})\frac{4P}{bh^{3}}l^{3} \end{array}$$

and

(59) $$\begin{array}{l} u=(\frac{d_{31}d_{31}}{\lambda_{33}}-s_{11})\frac{6P}{bh^{3}}x^{2}z-(\frac{d_{33}d_{31}}{\lambda_{33}}-s_{13}-s_{44}-\frac{d_{15}d_{31}}{\lambda_{33}})\frac{2P}{bh^{3}}z^{3}\\ -(\frac{d_{31}d_{31}}{\lambda_{33}}-s_{11})\frac{6P}{bh^{3}}l^{2}z-(\frac{d_{15}d_{31}}{\lambda_{33}}+s_{44})\frac{3P}{2bh}z \end{array}.$$

Similarly, substituting Equation (57) into Equations (A46), (A47), and (A48), the stress components can be written as

(60) $$\sigma_{x}=-\frac{12P}{bh^{3}}xz,$$

(61) $$\sigma_{z}=0,$$

and

(62) $$\tau_{zx}=\frac{6P}{bh^{3}}z^{2}-\frac{3P}{2bh}.$$

Substituting Equation (57) into Equations (A49) and (A50), the expressions of electric displacement components are

(63) $$D_{x}=(d_{15}+\frac{\lambda_{11}d_{31}}{\lambda_{33}})(\frac{6P}{bh^{3}}z^{2}-\frac{3P}{2bh})$$

and

(64) $$D_{z}=0.$$

By comparing Equations (58)–(64) with the expressions of displacement components, stress components, and electric displacement components in reference [17], it can be found that they are exactly the same, which indicates that the solution obtained here is correct. It should be mentioned that the structures studied in this paper and in reference [17] are both piezoelectric cantilever beams, but the structure in this paper is subjected to combined loads and the structure in reference [17] is subjected only to a concentrated force. In addition, non-dimensionalization is considered, the relevant experiments are carried out, and a new method called the multi-parameter perturbation method is given in this paper. These differences mentioned above constitute the advancements of this paper, compared with reference [17].

## 5. Experimental Verification

**Figure 2:**
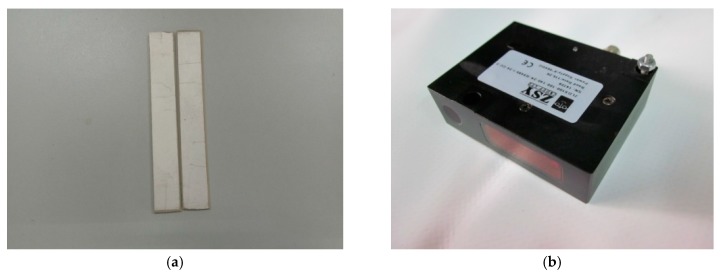
Scheme of experimental specimens and measuring instruments: (**a**) PbZrTiO_3_-5 (Generally abbreviated as PZT-5) piezoelectric ceramic specimens. (**b**) The non-contact laser displacement sensor.

**Figure 3:**
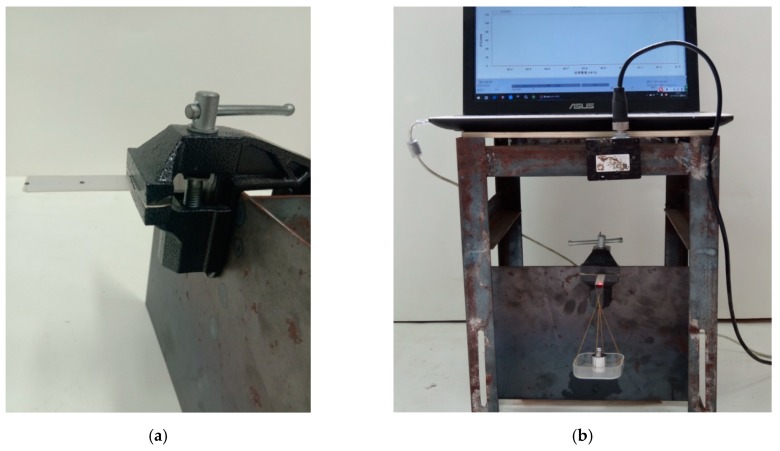
Scheme of experimental device: (**a**) The cantilever beam device. (**b**) The integral measuring device.

To further verify the validity of the theoretical solution presented here, we carry out the relevant experiments of piezoelectric cantilever beams. The mechanical model of the theoretical part is shown in Figure 1, it can be seen that it is a piezoelectric cantilever beam subjected to three kinds of loads. In the experiment, it is very difficult to apply these three kinds of loads at the same time. Therefore, we apply only the concentrated force at the cantilever end to carry out the experiments, that is, this experiment corresponds only to the case where the bending moment and the uniformly distributed load in the theoretical solution are zero. The details of the experiments are as follows. The main experimental equipments include a non-contact laser displacement sensor (ZSY Group Ltd, London, UK), a bench clamp (a cantilever beam clamping device), weights, and the ZLDS10X measuring software (ZSY Group Ltd, London, UK). The measuring range of the non-contact laser displacement sensor is 1 m, the accuracy is 0.01%, and the sampling frequency is 2 kHz. The experimental specimens consist of two groups of PbZrTiO_3_-5 (Generally abbreviated as PZT-5) piezoelectric ceramic sheets in which one group has piezoelectric properties and the other group has no piezoelectric properties. The size of the experimental specimens is 60 mm × 10 mm × 1 mm. The experimental specimen and non-contact laser displacement sensor are shown in Figure 2, the experimental device is shown in Figure 3, and the material constants are shown in Table 1.

The clamping length of the experimental specimens is 10 mm, therefore, the length of the piezoelectric cantilever beam is 50 mm. The deformations of the free end of piezoelectric cantilever beam are measured at the applied load 0.49 N, 0.98 N, and 1.96 N. The measured experimental data and theoretical calculation results are shown in Table 2 and Table 3, respectively. It should be noted that the self-weight of the piezoelectric cantilever beam is 0.0367 N, and the ratio of the self-weight to the minimum applied load is 0.075, which indicates that the self-weight of the piezoelectric cantilever beam is very small and thus may be ignored.

From Table 2, it can be seen that the theoretical results are in good agreement with the experimental results, and the relative errors under every level load are less than 15% allowed in engineering. This indicates that the analytical solution presented in this paper is reliable.

Table 3 shows that the deformation of the piezoelectric cantilever beam is smaller than the cantilever beam without piezoelectric properties. This means that the piezoelectric properties have a certain effect on the deformation of the piezoelectric cantilever beam, and its effect is, to a certain extent, hindering the deformation of the cantilever beam. This phenomenon can be explained by energy conservation. For piezoelectric cantilever beams, part of the work done by external forces is transformed into the elastic strain energy of piezoelectric cantilever beams, while the other part is transformed into the electric energy due to the existence of piezoelectric properties. For cantilever beams without piezoelectric properties, the work done by external forces is basically transformed into the elastic strain energy of cantilever beams. Therefore, the deformation of cantilever beams without piezoelectric properties is larger than that of cantilever beams with piezoelectric properties. The phenomenon mentioned above is commonly known as the piezoelectric stiffening effect peculiar to piezoelectric materials and structures.

## 6. Conclusions

In this study, we used a multi-parameter perturbation method to solve the bending deformation problem of piezoelectric cantilever beams under combined loads. And we compared the solution presented here with the existing solution from Yang and Liu [17] to validate the rationality of the presented solution. In addition, we carried out the related experiments of the piezoelectric cantilever beam, and compared the experimental results with the theoretical solution presented here, and also investigated the influence of the piezoelectric properties on the deformation of piezoelectric cantilever beams. The following main conclusions can be drawn.

(i) The theoretical results are in good agreement with the experimental results, which means that the analytical solution given in this paper is correct and the multi-parameter perturbation method is effective.

(ii) From the perturbation expansion, it is easy to find that the zero-order perturbation solution is a pure mechanical solution, in which the piezoelectric effect has not been incorporated. From the first-order, second-order, and higher order perturbation solutions, the piezoelectric effect is gradually reflected. This structural form of the multi-parameter perturbation solution presented here is beneficial to the analysis and understanding of the solved problem.

(iii) The deformation magnitude of a piezoelectric cantilever beam is smaller than that of a cantilever beam without piezoelectricity, due to the well-known piezoelectric stiffening effect.

Unfortunately, the numerical simulation for the physical system studied here has not been carried out in this study. In our previous study [34], we used ABAQUS software to simulate the problem of functionally graded piezoelectric cantilever beams with different properties in tension and compression. Similarly, the problem studied here may also be simulated by ABAQUS, which is our follow-up research. In summary, the multi-parameter perturbation method presented in this paper provides a new way to solve complex non-linear structural problems. The analytical solution of the bending problems of piezoelectric cantilever beams under combined loads can provide a theoretical basis and reference for the analysis and design of sensors or actuators made of piezoelectric polymers.

## Figures and Tables

**Table 1:** Physical properties of PZT-5 materials [33].

Elastic Constant (10^−12^ m^2^·N^−1^)					Piezoelectric Constant (10^−12^ C·N^−1^)			Dielectric Constant (10^−8^ F·m^−1^)	
$s_{11}^{0}$	$s_{12}^{0}$	$s_{13}^{0}$	$s_{33}^{0}$	$s_{44}^{0}$	$d_{31}^{0}$	$d_{33}^{0}$	$d_{15}^{0}$	$\lambda_{11}^{0}$	$\lambda_{33}^{0}$
16.4	−5.74	−7.22	18.8	47.5	−172	374	584	1.505	1.531

**Table 2:** Comparison of experimental data and theoretical calculation results.

Loads(N)	The Deformation of the Cantilever End		
	Experimental Data (mm)	Theoretical Results (mm)	Relative Errors (%)
0.49	0.4069	0.3545	12.87
0.98	0.7527	0.7089	5.82
1.96	1.6072	1.4178	11.79

**Table 3:** Comparison of deformation test results between piezoelectric cantilever beam and cantilever beam without piezoelectric properties.

Loads(N)	The Deformation of the Cantilever End		
	Piezoelectric Cantilever Beam (mm)	Cantilever Beam without Piezoelectric Properties (mm)	Difference (mm)
0.49	0.4069	0.5351	0.1282
0.98	0.7527	0.8463	0.0936
1.96	1.6072	1.9796	0.3724

## Appendix A

(1) The unknown functions $g_{i}^{0}(Z)$ and $f_{i}^{0}(Z)$ (i=1,2,3):

From Equations (27) and (33), we can obtain the unknown functions $g_{i}^{0}(Z)$ and $f_{i}^{0}(Z)$ (i=1,2,3),

(A1) $$\left\{ \begin{array}{l} g_{1}^{0}(Z)=B_{1}^{0}Z+B_{2}^{0}\\ g_{2}^{0}(Z)=B_{3}^{0}Z+B_{4}^{0}\\ g_{3}^{0}(Z)=-\frac{1}{3{\bar{\lambda }}_{33}}B_{1}^{0}Z^{3}-\frac{1}{{\bar{\lambda }}_{33}}B_{2}^{0}Z^{2}+B_{5}^{0}Z+B_{6}^{0} \end{array}\right.$$

and

(A2) $$\left\{ \begin{array}{l} f_{1}^{0}(Z)=\frac{1}{6}C_{1}^{0}Z^{3}+\frac{1}{2}C_{2}^{0}Z^{2}+C_{3}^{0}Z+C_{4}^{0}\\ f_{2}^{0}(Z)=\frac{1}{6}C_{5}^{0}Z^{3}+\frac{1}{2}C_{6}^{0}Z^{2}+C_{7}^{0}Z+C_{8}^{0}\\ f_{3}^{0}(Z)=-\frac{1}{120}(2S_{13}+S_{44})C_{1}^{0}Z^{5}-\frac{1}{24}(2S_{13}+S_{44})C_{2}^{0}Z^{4}\\ +\frac{1}{6}C_{9}^{0}Z^{3}+\frac{1}{2}C_{10}^{0}Z^{2}+C_{11}^{0}Z+C_{12}^{0} \end{array}\right.,$$

where $B_{i}^{0}$ (i=1,2,3,…,6) and $C_{i}^{0}$ (i=1,2,3,…,12) are undetermined constants which can be determined by Equations (28)–(32),

(A3) $$\begin{array}{l} C_{1}^{0}=-12\bar{q},C_{2}^{0}=0,C_{3}^{0}=\frac{3}{2}\bar{q},C_{4}^{0}=-\frac{\bar{q}}{2},C_{5}^{0}=-\frac{12\bar{P}}{\bar{b}},C_{6}^{0}=0,\\ C_{7}^{0}=\frac{3}{2}\frac{\bar{P}}{\bar{b}},C_{9}^{0}=\frac{12\bar{M}}{\bar{b}}-\frac{3}{10}(2S_{13}+S_{44})\bar{q},C_{10}^{0}=0 \end{array},$$

(A4) $$B_{1}^{0}=0,B_{2}^{0}=0,B_{3}^{0}=0,B_{5}^{0}=0.$$

(2) The unknown functions $g_{i}^{Ι}(Z)\;\mathrm{and}\;f_{i}^{Ι}(Z)\;(i=1,2,3,\ldots ,9)\text{:}$

From Equations (34)–(36) and (42), we can obtain the unknown functions $g_{i}^{Ι}(Z)$ and $f_{i}^{Ι}(Z)$ (i=1,2,3,…,9),

(A5) $$\left\{ \begin{array}{l} g_{1}^{Ι}(Z)=\frac{1}{4{\bar{\lambda }}_{33}}C_{1}^{0}Z^{2}+B_{1}^{Ι}Z+B_{2}^{Ι}\\ g_{2}^{Ι}(Z)=\frac{1}{2{\bar{\lambda }}_{33}}C_{5}^{0}Z^{2}+B_{3}^{Ι}Z+B_{4}^{Ι}\\ g_{3}^{Ι}(Z)=-\frac{1}{24{\left({\bar{\lambda }}_{33}\right)}^{2}}C_{1}^{0}Z^{4}-\frac{1}{3{\bar{\lambda }}_{33}}B_{1}^{Ι}Z^{3}-\frac{1}{{\bar{\lambda }}_{33}}B_{2}^{Ι}Z^{2}-\frac{\left(2S_{13}+S_{44}\right)}{24{\bar{\lambda }}_{33}}C_{1}^{0}Z^{4}\\ -\frac{\left(2S_{13}+S_{44}\right)}{6{\bar{\lambda }}_{33}}C_{2}^{0}Z^{3}+\frac{1}{2{\bar{\lambda }}_{33}}C_{9}^{0}Z^{2}+B_{5}^{Ι}Z+B_{6}^{Ι}\\ g_{6}^{Ι}(Z)=-\frac{1}{3{\bar{\lambda }}_{33}}B_{7}^{Ι}Z^{3}-\frac{1}{{\bar{\lambda }}_{33}}B_{8}^{Ι}Z^{2}+\frac{C_{1}^{0}}{24{\bar{\lambda }}_{33}}Z^{4}\\ +\frac{C_{2}^{0}}{6{\bar{\lambda }}_{33}}Z^{3}+\frac{C_{3}^{0}}{2{\bar{\lambda }}_{33}}Z^{2}+B_{11}^{Ι}Z+B_{12}^{Ι}\\ g_{9}^{Ι}(Z)=-\frac{1}{3{\bar{\lambda }}_{33}}B_{13}^{Ι}Z^{3}-\frac{1}{{\bar{\lambda }}_{33}}B_{14}^{Ι}Z^{2}-\frac{C_{1}^{0}}{24{\bar{\lambda }}_{33}}Z^{4}-\frac{C_{2}^{0}}{6{\bar{\lambda }}_{33}}Z^{3}-\frac{C_{3}^{0}}{2{\bar{\lambda }}_{33}}Z^{2}+B_{17}^{Ι}Z+B_{18}^{Ι} \end{array}\right.,$$

(A6) $$g_{i}^{Ι}(Z)=B_{2i-1}^{Ι}Z+B_{2i}^{Ι}(i=4,5,7,8),$$

(A7) $$f_{i}^{Ι}(Z)=\frac{1}{6}C_{4i-3}^{Ι}Z^{3}+\frac{1}{2}C_{4i-2}^{Ι}Z^{2}+C_{4i-1}^{Ι}Z+C_{4i}^{Ι}(i=1,2,4,5,7,8),$$

and

(A8) $$\left\{ \begin{array}{l} f_{3}^{Ι}(Z)=-\frac{\left(2S_{13}+S_{44}\right)}{120}C_{1}^{Ι}Z^{5}-\frac{\left(2S_{13}+S_{44}\right)}{24}C_{2}^{Ι}Z^{4}-\frac{1}{12{\bar{\lambda }}_{33}}B_{1}^{0}Z^{4}\\ +\frac{1}{6}C_{9}^{Ι}Z^{3}+\frac{1}{2}C_{10}^{Ι}Z^{2}+C_{11}^{Ι}Z+C_{12}^{Ι}\\ f_{6}^{Ι}(Z)=-\frac{\left(2S_{13}+S_{44}\right)}{120}C_{13}^{Ι}Z^{5}-\frac{\left(2S_{13}+S_{44}\right)}{24}C_{14}^{Ι}Z^{4}+\frac{1}{12}B_{1}^{0}Z^{4}\\ +\frac{1}{6}C_{21}^{Ι}Z^{3}+\frac{1}{2}C_{22}^{Ι}Z^{2}+C_{23}^{Ι}Z+C_{24}^{Ι}\\ f_{9}^{Ι}(Z)=-\frac{\left(2S_{13}+S_{44}\right)}{120}C_{25}^{Ι}Z^{5}-\frac{\left(2S_{13}+S_{44}\right)}{24}C_{26}^{Ι}Z^{4}-\frac{B_{1}^{0}}{12}Z^{4}\\ +\frac{1}{6}C_{33}^{Ι}Z^{3}+\frac{1}{2}C_{34}^{Ι}Z^{2}+C_{35}^{Ι}Z+C_{36}^{Ι} \end{array}\right..$$

where $B_{i}^{Ι}$ (i=1,2,3,…,18) and $C_{i}^{Ι}$ (i=1,2,3,…,36) are undetermined constants which can be determined by Equations (37)–(41),

(A9) $$C_{1}^{Ι}=0,C_{2}^{Ι}=0,C_{3}^{Ι}=0,C_{4}^{Ι}=0,C_{5}^{Ι}=0,C_{6}^{Ι}=0,C_{7}^{Ι}=0,C_{9}^{Ι}=0,C_{10}^{Ι}=\frac{1}{12{\bar{\lambda }}_{33}}B_{1}^{0},$$

(A10) $$B_{1}^{Ι}=\frac{C_{2}^{0}}{2{\bar{\lambda }}_{33}},B_{2}^{Ι}=-\frac{C_{1}^{0}}{48{\bar{\lambda }}_{33}},B_{3}^{Ι}=\frac{C_{6}^{0}}{{\bar{\lambda }}_{33}},B_{4}^{Ι}=-\frac{C_{5}^{0}}{24{\bar{\lambda }}_{33}},B_{5}^{Ι}=\frac{C_{10}^{0}}{{\bar{\lambda }}_{33}}+\frac{C_{2}^{0}}{8{\left({\bar{\lambda }}_{33}\right)}^{2}},$$

(A11) $$C_{13}^{Ι}=0,C_{14}^{Ι}=0,C_{15}^{Ι}=0,C_{16}^{Ι}=0,C_{17}^{Ι}=0,C_{18}^{Ι}=0,C_{19}^{Ι}=0,C_{21}^{Ι}=0,C_{22}^{Ι}=-\frac{1}{12}B_{1}^{0},$$

(A12) $$B_{7}^{Ι}=0,B_{8}^{Ι}=0,B_{9}^{Ι}=0,B_{10}^{Ι}=0,B_{11}^{Ι}=\frac{C_{4}^{0}}{{\bar{\lambda }}_{33}},$$

(A13) $$C_{25}^{Ι}=0,C_{26}^{Ι}=0,C_{27}^{Ι}=0,C_{28}^{Ι}=0,C_{29}^{Ι}=0,C_{30}^{Ι}=0,C_{31}^{Ι}=0,C_{33}^{Ι}=0,C_{34}^{Ι}=\frac{B_{1}^{0}}{12},$$

(A14) $$B_{13}^{Ι}=0,B_{14}^{Ι}=-\frac{1}{48}C_{1}^{0}-\frac{1}{2}C_{3}^{0},B_{15}^{Ι}=0,B_{16}^{Ι}=-\frac{1}{24}C_{5}^{0}-C_{7}^{0},B_{17}^{Ι}=\frac{C_{2}^{0}}{8{\bar{\lambda }}_{33}}.$$

(3) The unknown functions $g_{i}^{\mathrm{ΙΙ}}(Z)\mathrm{and}\;f_{i}^{\mathrm{ΙΙ}}(Z)\;(i=1,2,3,\ldots ,18)\text{:}$

From Equations (43)–(48) and (54), we may obtain the unknown functions of $g_{i}^{\mathrm{ΙΙ}}(Z)$ and $f_{i}^{\mathrm{ΙΙ}}(Z)$ (i=1,2,3,…,18),

(A15) $$\left\{ \begin{array}{l} g_{i}^{\mathrm{ΙΙ}}(Z)=K_{i}Z^{2}+B_{2i-1}^{\mathrm{ΙΙ}}Z+B_{2i}^{\mathrm{ΙΙ}}(i=1,2,10,11,13,14)\\ g_{i}^{\mathrm{ΙΙ}}(Z)=B_{2i-1}^{\mathrm{ΙΙ}}Z+B_{2i}^{\mathrm{ΙΙ}}(i=4,5,7,8,16,17) \end{array}\right.,$$

(A16) $$\left\{ \begin{array}{l} g_{3}^{\mathrm{ΙΙ}}(Z)=-\frac{C_{1}^{Ι}}{24{\left({\bar{\lambda }}_{33}\right)}^{2}}Z^{4}-\frac{1}{3{\bar{\lambda }}_{33}}B_{1}^{\mathrm{ΙΙ}}Z^{3}-\frac{1}{{\bar{\lambda }}_{33}}B_{2}^{\mathrm{ΙΙ}}Z^{2}-\frac{\left(2S_{13}+S_{44}\right)}{24{\bar{\lambda }}_{33}}C_{1}^{Ι}Z^{4}\\ -\frac{\left(2S_{13}+S_{44}\right)}{6{\bar{\lambda }}_{33}}C_{2}^{Ι}Z^{3}-\frac{1}{3{\left({\bar{\lambda }}_{33}\right)}^{2}}B_{1}^{0}Z^{3}+\frac{1}{2{\bar{\lambda }}_{33}}C_{9}^{Ι}Z^{2}+B_{5}^{\mathrm{ΙΙ}}Z+B_{6}^{\mathrm{ΙΙ}}\\ g_{6}^{\mathrm{ΙΙ}}(Z)=-\frac{1}{3{\bar{\lambda }}_{33}}B_{7}^{\mathrm{ΙΙ}}Z^{3}-\frac{1}{{\bar{\lambda }}_{33}}B_{8}^{\mathrm{ΙΙ}}Z^{2}+\frac{1}{24{\bar{\lambda }}_{33}}C_{13}^{Ι}Z^{4}+\frac{1}{6{\bar{\lambda }}_{33}}C_{14}^{Ι}Z^{3}+\frac{1}{2{\bar{\lambda }}_{33}}C_{15}^{Ι}Z^{2}+B_{11}^{\mathrm{ΙΙ}}Z+B_{12}^{\mathrm{ΙΙ}}\\ g_{9}^{\mathrm{ΙΙ}}(Z)=-\frac{1}{3{\bar{\lambda }}_{33}}B_{13}^{\mathrm{ΙΙ}}Z^{3}-\frac{1}{{\bar{\lambda }}_{33}}B_{14}^{\mathrm{ΙΙ}}Z^{2}-\frac{1}{24{\bar{\lambda }}_{33}}C_{25}^{Ι}Z^{4}-\frac{C_{26}^{Ι}}{6{\bar{\lambda }}_{33}}Z^{3}-\frac{C_{27}^{Ι}}{2{\bar{\lambda }}_{33}}Z^{2}+B_{17}^{\mathrm{ΙΙ}}Z+B_{18}^{\mathrm{ΙΙ}}\\ g_{12}^{\mathrm{ΙΙ}}(Z)=-\frac{C_{13}^{Ι}}{24{\left({\bar{\lambda }}_{33}\right)}^{2}}Z^{4}-\frac{1}{3{\bar{\lambda }}_{33}}B_{19}^{\mathrm{ΙΙ}}Z^{3}-\frac{1}{{\bar{\lambda }}_{33}}B_{20}^{\mathrm{ΙΙ}}Z^{2}+\frac{1}{24{\bar{\lambda }}_{33}}C_{1}^{Ι}Z^{4}+\frac{1}{6{\bar{\lambda }}_{33}}C_{2}^{Ι}Z^{3}+\frac{1}{2{\bar{\lambda }}_{33}}C_{3}^{Ι}Z^{2}\\ -\frac{\left(2S_{13}+S_{44}\right)}{24{\bar{\lambda }}_{33}}C_{13}^{Ι}Z^{4}-\frac{\left(2S_{13}+S_{44}\right)}{6{\bar{\lambda }}_{33}}C_{14}^{Ι}Z^{3}+\frac{1}{3{\bar{\lambda }}_{33}}B_{1}^{0}Z^{3}+\frac{1}{2{\bar{\lambda }}_{33}}C_{21}^{Ι}Z^{2}+B_{23}^{\mathrm{ΙΙ}}Z+B_{24}^{\mathrm{ΙΙ}}\\ g_{15}^{\mathrm{ΙΙ}}(Z)=-\frac{C_{25}^{Ι}}{24{\left({\bar{\lambda }}_{33}\right)}^{2}}Z^{4}-\frac{1}{3{\bar{\lambda }}_{33}}B_{25}^{\mathrm{ΙΙ}}Z^{3}-\frac{1}{{\bar{\lambda }}_{33}}B_{26}^{\mathrm{ΙΙ}}Z^{2}-\frac{\left(2S_{13}+S_{44}\right)}{24{\bar{\lambda }}_{33}}C_{25}^{Ι}Z^{4}-\frac{\left(2S_{13}+S_{44}\right)}{6{\bar{\lambda }}_{33}}C_{26}^{Ι}Z^{3}\\ -\frac{1}{3{\bar{\lambda }}_{33}}B_{1}^{0}Z^{3}+\frac{C_{33}^{Ι}}{2{\bar{\lambda }}_{33}}Z^{2}-\frac{1}{24{\bar{\lambda }}_{33}}C_{1}^{Ι}Z^{4}-\frac{C_{2}^{Ι}}{6{\bar{\lambda }}_{33}}Z^{3}-\frac{C_{3}^{Ι}}{2{\bar{\lambda }}_{33}}Z^{2}+B_{29}^{\mathrm{ΙΙ}}Z+B_{30}^{\mathrm{ΙΙ}}\\ g_{18}^{\mathrm{ΙΙ}}(Z)=-\frac{1}{3{\bar{\lambda }}_{33}}B_{31}^{\mathrm{ΙΙ}}Z^{3}-\frac{1}{{\bar{\lambda }}_{33}}B_{32}^{\mathrm{ΙΙ}}Z^{2}+\frac{C_{25}^{Ι}}{24{\bar{\lambda }}_{33}}Z^{4}+\frac{C_{26}^{Ι}}{6{\bar{\lambda }}_{33}}Z^{3}+\frac{C_{27}^{Ι}}{2{\bar{\lambda }}_{33}}Z^{2}\\ -\frac{C_{13}^{Ι}}{24{\bar{\lambda }}_{33}}Z^{4}-\frac{C_{14}^{Ι}}{6{\bar{\lambda }}_{33}}Z^{3}-\frac{C_{15}^{Ι}}{2{\bar{\lambda }}_{33}}Z^{2}+B_{35}^{\mathrm{ΙΙ}}Z+B_{36}^{\mathrm{ΙΙ}} \end{array}\right.,$$

(A17) $$\begin{array}{l} f_{i}^{\mathrm{ΙΙ}}(Z)=\frac{1}{6}C_{4i-3}^{\mathrm{ΙΙ}}Z^{3}+\frac{1}{2}C_{4i-2}^{\mathrm{ΙΙ}}Z^{2}+C_{4i-1}^{\mathrm{ΙΙ}}Z\\ +C_{4i}^{\mathrm{ΙΙ}}(i=1,2,4,5,7,8,10,11,13,14,16,17) \end{array},$$

(A18) $$\left\{ \begin{array}{l} f_{3}^{\mathrm{ΙΙ}}(Z)=-\frac{\left(2S_{13}+S_{44}\right)}{120}C_{1}^{\mathrm{ΙΙ}}Z^{5}-\frac{\left(2S_{13}+S_{44}\right)}{24}C_{2}^{\mathrm{ΙΙ}}Z^{4}-\frac{C_{1}^{0}}{120{\left({\bar{\lambda }}_{33}\right)}^{2}}Z^{5}-\frac{1}{12{\bar{\lambda }}_{33}}B_{1}^{Ι}Z^{4}\\ -\frac{\left(2S_{13}+S_{44}\right)}{120{\bar{\lambda }}_{33}}C_{1}^{0}Z^{5}-\frac{\left(2S_{13}+S_{44}\right)}{24{\bar{\lambda }}_{33}}C_{2}^{0}Z^{4}+\frac{1}{6}C_{9}^{\mathrm{ΙΙ}}Z^{3}+\frac{1}{2}C_{10}^{\mathrm{ΙΙ}}Z^{2}+C_{11}^{\mathrm{ΙΙ}}Z+C_{12}^{\mathrm{ΙΙ}}\\ f_{6}^{\mathrm{ΙΙ}}(Z)=-\frac{\left(2S_{13}+S_{44}\right)}{120}C_{13}^{\mathrm{ΙΙ}}Z^{5}-\frac{\left(2S_{13}+S_{44}\right)}{24}C_{14}^{\mathrm{ΙΙ}}Z^{4}+\frac{1}{12}B_{7}^{Ι}Z^{4}\\ +\frac{1}{6}C_{21}^{\mathrm{ΙΙ}}Z^{3}+\frac{1}{2}C_{22}^{\mathrm{ΙΙ}}Z^{2}+C_{23}^{\mathrm{ΙΙ}}Z+C_{24}^{\mathrm{ΙΙ}}\\ f_{9}^{\mathrm{ΙΙ}}(Z)=-\frac{\left(2S_{13}+S_{44}\right)}{120}C_{25}^{\mathrm{ΙΙ}}Z^{5}-\frac{\left(2S_{13}+S_{44}\right)}{24}C_{26}^{\mathrm{ΙΙ}}Z^{4}-\frac{1}{12}B_{13}^{Ι}Z^{4}\\ +\frac{1}{6}C_{33}^{\mathrm{ΙΙ}}Z^{3}+\frac{1}{2}C_{34}^{\mathrm{ΙΙ}}Z^{2}+C_{35}^{\mathrm{ΙΙ}}Z+C_{36}^{\mathrm{ΙΙ}}\\ f_{12}^{\mathrm{ΙΙ}}(Z)=-\frac{\left(2S_{13}+S_{44}\right)}{120}C_{37}^{\mathrm{ΙΙ}}Z^{5}-\frac{\left(2S_{13}+S_{44}\right)}{24}C_{38}^{\mathrm{ΙΙ}}Z^{4}-\frac{1}{12{\bar{\lambda }}_{33}}B_{7}^{Ι}Z^{4}+\frac{C_{1}^{0}}{60{\bar{\lambda }}_{33}}Z^{5}\\ +\frac{C_{2}^{0}}{24{\bar{\lambda }}_{33}}Z^{4}+\frac{1}{12}B_{1}^{Ι}Z^{4}+\frac{1}{6}C_{45}^{\mathrm{ΙΙ}}Z^{3}+\frac{1}{2}C_{46}^{\mathrm{ΙΙ}}Z^{2}+C_{47}^{\mathrm{ΙΙ}}Z+C_{48}^{\mathrm{ΙΙ}}\\ f_{15}^{\mathrm{ΙΙ}}(Z)=-\frac{\left(2S_{13}+S_{44}\right)}{120}C_{49}^{\mathrm{ΙΙ}}Z^{5}-\frac{\left(2S_{13}+S_{44}\right)}{24}C_{50}^{\mathrm{ΙΙ}}Z^{4}-\frac{1}{12{\bar{\lambda }}_{33}}B_{13}^{Ι}Z^{4}-\frac{C_{1}^{0}}{60{\bar{\lambda }}_{33}}Z^{5}\\ -\frac{C_{2}^{0}}{24{\bar{\lambda }}_{33}}Z^{4}-\frac{1}{12}B_{1}^{Ι}Z^{4}+\frac{1}{6}C_{57}^{\mathrm{ΙΙ}}Z^{3}+\frac{1}{2}C_{58}^{\mathrm{ΙΙ}}Z^{2}+C_{59}^{\mathrm{ΙΙ}}Z+C_{60}^{\mathrm{ΙΙ}}\\ f_{18}^{\mathrm{ΙΙ}}(Z)=-\frac{\left(2S_{13}+S_{44}\right)}{120}C_{61}^{\mathrm{ΙΙ}}Z^{5}-\frac{\left(2S_{13}+S_{44}\right)}{24}C_{62}^{\mathrm{ΙΙ}}Z^{4}+\frac{1}{12}B_{13}^{Ι}Z^{4}\\ -\frac{1}{12}B_{7}^{Ι}Z^{4}+\frac{1}{6}C_{69}^{\mathrm{ΙΙ}}Z^{3}+\frac{1}{2}C_{70}^{\mathrm{ΙΙ}}Z^{2}+C_{71}^{\mathrm{ΙΙ}}Z+C_{72}^{\mathrm{ΙΙ}} \end{array}\right.,$$

and

(A19) $$K_{1}=\frac{C_{1}^{Ι}}{4{\bar{\lambda }}_{33}},K_{2}=\frac{C_{5}^{Ι}}{2{\bar{\lambda }}_{33}},K_{10}=\frac{C_{13}^{Ι}}{4{\bar{\lambda }}_{33}},K_{11}=\frac{C_{17}^{Ι}}{2{\bar{\lambda }}_{33}},K_{13}=\frac{C_{25}^{Ι}}{4{\bar{\lambda }}_{33}},K_{14}=\frac{C_{29}^{Ι}}{2{\bar{\lambda }}_{33}},$$

where $B_{i}^{\mathrm{ΙΙ}}$ (i=1,2,3,…,36) and $C_{i}^{\mathrm{ΙΙ}}$ (i=1,2,3,…,72) are undetermined constants which can be determined by Equations (49)–(53),

(A20) $$\begin{array}{l} C_{1}^{\mathrm{ΙΙ}}=0,C_{2}^{\mathrm{ΙΙ}}=0,C_{3}^{\mathrm{ΙΙ}}=0,C_{4}^{\mathrm{ΙΙ}}=0,C_{5}^{\mathrm{ΙΙ}}=0,C_{6}^{\mathrm{ΙΙ}}=0,C_{7}^{\mathrm{ΙΙ}}=0,\\ C_{9}^{\mathrm{ΙΙ}}=\frac{1}{40{\left({\bar{\lambda }}_{33}\right)}^{2}}C_{1}^{0}+\frac{\left(2S_{13}+S_{44}\right)}{40{\bar{\lambda }}_{33}}C_{1}^{0},C_{10}^{\mathrm{ΙΙ}}=\frac{1}{12{\bar{\lambda }}_{33}}B_{1}^{Ι}+\frac{\left(2S_{13}+S_{44}\right)}{24{\bar{\lambda }}_{33}}C_{2}^{0} \end{array},$$

(A21) $$B_{1}^{\mathrm{ΙΙ}}=\frac{C_{2}^{Ι}}{2{\bar{\lambda }}_{33}},B_{2}^{\mathrm{ΙΙ}}=-\frac{C_{1}^{Ι}}{48{\bar{\lambda }}_{33}},B_{3}^{\mathrm{ΙΙ}}=\frac{C_{6}^{Ι}}{{\bar{\lambda }}_{33}},B_{4}^{\mathrm{ΙΙ}}=-\frac{C_{5}^{Ι}}{24{\bar{\lambda }}_{33}},B_{5}^{\mathrm{ΙΙ}}=\frac{C_{10}^{Ι}}{{\bar{\lambda }}_{33}}+\frac{C_{2}^{Ι}}{8{\left({\bar{\lambda }}_{33}\right)}^{2}},$$

(A22) $$C_{13}^{\mathrm{ΙΙ}}=0,C_{14}^{\mathrm{ΙΙ}}=0,C_{15}^{\mathrm{ΙΙ}}=0,C_{16}^{\mathrm{ΙΙ}}=0,C_{17}^{\mathrm{ΙΙ}}=0,C_{18}^{\mathrm{ΙΙ}}=0,C_{19}^{\mathrm{ΙΙ}}=0,C_{21}^{\mathrm{ΙΙ}}=0,C_{22}^{\mathrm{ΙΙ}}=-\frac{1}{12}B_{7}^{Ι},$$

(A23) $$B_{7}^{\mathrm{ΙΙ}}=0,B_{8}^{\mathrm{ΙΙ}}=0,B_{9}^{\mathrm{ΙΙ}}=0,B_{10}^{\mathrm{ΙΙ}}=0,B_{11}^{\mathrm{ΙΙ}}=\frac{C_{16}^{Ι}}{{\bar{\lambda }}_{33}},$$

(A24) $$C_{25}^{\mathrm{ΙΙ}}=0,C_{26}^{\mathrm{ΙΙ}}=0,C_{27}^{\mathrm{ΙΙ}}=0,C_{28}^{\mathrm{ΙΙ}}=0,C_{29}^{\mathrm{ΙΙ}}=0,C_{30}^{\mathrm{ΙΙ}}=0,C_{31}^{\mathrm{ΙΙ}}=0,C_{33}^{\mathrm{ΙΙ}}=0,C_{34}^{\mathrm{ΙΙ}}=\frac{1}{12}B_{13}^{Ι},$$

(A25) $$B_{13}^{\mathrm{ΙΙ}}=0,B_{14}^{\mathrm{ΙΙ}}=-\frac{1}{48}C_{25}^{Ι}-\frac{1}{2}C_{27}^{Ι},B_{15}^{\mathrm{ΙΙ}}=0,B_{16}^{\mathrm{ΙΙ}}=-\frac{1}{24}C_{29}^{Ι}-C_{31}^{Ι},B_{17}^{\mathrm{ΙΙ}}=\frac{C_{26}^{Ι}}{8{\bar{\lambda }}_{33}},$$

(A26) $$\begin{array}{l} C_{37}^{\mathrm{ΙΙ}}=0,C_{38}^{\mathrm{ΙΙ}}=0,C_{39}^{\mathrm{ΙΙ}}=0,C_{40}^{\mathrm{ΙΙ}}=0,C_{41}^{\mathrm{ΙΙ}}=0,C_{42}^{\mathrm{ΙΙ}}=0,C_{43}^{\mathrm{ΙΙ}}=0,\\ C_{45}^{\mathrm{ΙΙ}}=-\frac{C_{1}^{0}}{20{\bar{\lambda }}_{33}},C_{46}^{\mathrm{ΙΙ}}=\frac{1}{12{\bar{\lambda }}_{33}}B_{7}^{Ι}-\frac{C_{2}^{0}}{24{\bar{\lambda }}_{33}}-\frac{1}{12}B_{1}^{Ι} \end{array},$$

(A27) $$B_{19}^{\mathrm{ΙΙ}}=\frac{C_{14}^{Ι}}{2{\bar{\lambda }}_{33}},B_{20}^{\mathrm{ΙΙ}}=-\frac{C_{13}^{Ι}}{48{\bar{\lambda }}_{33}},B_{21}^{\mathrm{ΙΙ}}=\frac{C_{18}^{Ι}}{{\bar{\lambda }}_{33}},B_{22}^{\mathrm{ΙΙ}}=-\frac{C_{17}^{Ι}}{24{\bar{\lambda }}_{33}},B_{23}^{\mathrm{ΙΙ}}=\frac{C_{22}^{Ι}}{{\bar{\lambda }}_{33}}+\frac{C_{4}^{Ι}}{{\bar{\lambda }}_{33}}+\frac{C_{14}^{Ι}}{8{\left({\bar{\lambda }}_{33}\right)}^{2}},$$

(A28) $$\begin{array}{l} C_{49}^{\mathrm{ΙΙ}}=0,C_{50}^{\mathrm{ΙΙ}}=0,C_{51}^{\mathrm{ΙΙ}}=0,C_{52}^{\mathrm{ΙΙ}}=0,C_{53}^{\mathrm{ΙΙ}}=0,C_{54}^{\mathrm{ΙΙ}}=0,C_{55}^{\mathrm{ΙΙ}}=0,\\ C_{57}^{\mathrm{ΙΙ}}=\frac{C_{1}^{0}}{20{\bar{\lambda }}_{33}},C_{58}^{\mathrm{ΙΙ}}=\frac{1}{12{\bar{\lambda }}_{33}}B_{13}^{Ι}+\frac{C_{2}^{0}}{24{\bar{\lambda }}_{33}}+\frac{1}{12}B_{1}^{Ι} \end{array},$$

(A29) $$\begin{array}{l} B_{25}^{\mathrm{ΙΙ}}=\frac{C_{26}^{Ι}}{2{\bar{\lambda }}_{33}},B_{26}^{\mathrm{ΙΙ}}=-\frac{C_{1}^{Ι}}{48}-\frac{C_{3}^{Ι}}{2}-\frac{C_{25}^{Ι}}{48{\bar{\lambda }}_{33}},B_{27}^{\mathrm{ΙΙ}}=\frac{C_{30}^{Ι}}{{\bar{\lambda }}_{33}}\\ B_{28}^{\mathrm{ΙΙ}}=-\frac{1}{24}C_{5}^{Ι}-C_{7}^{Ι}-\frac{C_{29}^{Ι}}{24{\bar{\lambda }}_{33}},B_{29}^{\mathrm{ΙΙ}}=\frac{C_{34}^{Ι}}{{\bar{\lambda }}_{33}}+\frac{C_{26}^{Ι}}{8{\left({\bar{\lambda }}_{33}\right)}^{2}}+\frac{C_{2}^{Ι}}{8{\bar{\lambda }}_{33}} \end{array},$$

(A30) $$\begin{array}{l} C_{61}^{\mathrm{ΙΙ}}=0,C_{62}^{\mathrm{ΙΙ}}=0,C_{63}^{\mathrm{ΙΙ}}=0,C_{64}^{\mathrm{ΙΙ}}=0,C_{65}^{\mathrm{ΙΙ}}=0,C_{66}^{\mathrm{ΙΙ}}=0,C_{67}^{\mathrm{ΙΙ}}=0,\\ C_{69}^{\mathrm{ΙΙ}}=0,C_{70}^{\mathrm{ΙΙ}}=-\frac{1}{12}B_{13}^{Ι}+\frac{1}{12}B_{7}^{Ι} \end{array},$$

and

(A31) $$\begin{array}{l} B_{31}^{\mathrm{ΙΙ}}=0,B_{32}^{\mathrm{ΙΙ}}=-\frac{1}{48}C_{13}^{Ι}-\frac{1}{2}C_{15}^{Ι},B_{33}^{\mathrm{ΙΙ}}=0,\\ B_{34}^{\mathrm{ΙΙ}}=-\frac{1}{24}C_{17}^{Ι}-C_{19}^{Ι},B_{35}^{\mathrm{ΙΙ}}=\frac{C_{28}^{Ι}}{{\bar{\lambda }}_{33}}+\frac{C_{14}^{Ι}}{8{\bar{\lambda }}_{33}} \end{array}.$$

## Appendix B

From Equations (3)–(4), (16), and (55)–(56), we have

(A32) $$\begin{array}{l} \frac{\partial \bar{w}}{\partial Z}=S_{13}\frac{\partial^{2}\bar{U}}{\partial Z^{2}}+S_{33}\frac{\partial^{2}U}{\partial X^{2}}-{\bar{d}}_{33}\frac{\partial \Phi }{\partial Z}\\ =-6S_{13}\bar{q}X^{2}Z-\frac{12}{\bar{b}}S_{13}\bar{P}XZ+2(2S_{13}+S_{44})S_{13}\bar{q}Z^{3}-\frac{3(2S_{13}+S_{44})}{10}S_{13}\bar{q}Z\\ +\frac{12}{\bar{b}}S_{13}\bar{M}Z+S_{13}{\left({\bar{d}}_{31}\right)}^{2}[\frac{2}{{\left({\bar{\lambda }}_{33}\right)}^{2}}\bar{q}Z^{3}+\frac{2(2S_{13}+S_{44})}{{\bar{\lambda }}_{33}}\bar{q}Z^{3}-\frac{3}{10{\left({\bar{\lambda }}_{33}\right)}^{2}}\bar{q}Z-\frac{3(2S_{13}+S_{44})}{10{\bar{\lambda }}_{33}}\bar{q}Z]\\ +S_{13}{\bar{d}}_{31}{\bar{d}}_{33}(-\frac{4}{{\bar{\lambda }}_{33}}\bar{q}Z^{3}+\frac{3}{5{\bar{\lambda }}_{33}}\bar{q}Z)+S_{13}{\bar{d}}_{31}{\bar{d}}_{15}(\frac{4}{{\bar{\lambda }}_{33}}\bar{q}Z^{3}-\frac{3}{5{\bar{\lambda }}_{33}}\bar{q}Z)-2S_{33}\bar{q}Z^{3}+\frac{3}{2}S_{33}\bar{q}Z-S_{33}\frac{\bar{q}}{2}\\ -{\bar{d}}_{33}{\bar{d}}_{31}[-\frac{6}{{\bar{\lambda }}_{33}}\bar{q}X^{2}Z-\frac{12}{\bar{b}{\bar{\lambda }}_{33}}\bar{P}XZ+\frac{2}{{\left({\bar{\lambda }}_{33}\right)}^{2}}\bar{q}Z^{3}-\frac{1}{2{\left({\bar{\lambda }}_{33}\right)}^{2}}\bar{q}Z+\frac{2(2S_{13}+S_{44})}{{\bar{\lambda }}_{33}}\bar{q}Z^{3}+\frac{12}{\bar{b}{\bar{\lambda }}_{33}}\bar{M}Z\\ -\frac{3(2S_{13}+S_{44})}{10{\bar{\lambda }}_{33}}\bar{q}Z]-{\bar{d}}_{33}{\bar{d}}_{33}[-\frac{2}{{\bar{\lambda }}_{33}}\bar{q}Z^{3}+\frac{3}{2{\bar{\lambda }}_{33}}\bar{q}Z-\frac{1}{2{\bar{\lambda }}_{33}}\bar{q}]-{\bar{d}}_{33}{\bar{d}}_{15}[\frac{1}{{\bar{\lambda }}_{33}}\bar{q}Z+\frac{2}{{\bar{\lambda }}_{33}}\bar{q}Z^{3}-\frac{3}{2{\bar{\lambda }}_{33}}\bar{q}Z] \end{array}$$

and

(A33) $$\begin{array}{l} \frac{\partial \bar{u}}{\partial X}=\frac{\partial^{2}\bar{U}}{\partial Z^{2}}+S_{13}\frac{\partial^{2}\bar{U}}{\partial X^{2}}-{\bar{d}}_{31}\frac{\partial \Phi }{\partial Z}\\ =-6\bar{q}X^{2}Z-\frac{12}{\bar{b}}\bar{P}XZ+2(2S_{13}+S_{44})\bar{q}Z^{3}+\frac{12}{\bar{b}}\bar{M}Z-\frac{3(2S_{13}+S_{44})}{10}\bar{q}Z+{\left({\bar{d}}_{31}\right)}^{2}[\frac{2}{{\left({\bar{\lambda }}_{33}\right)}^{2}}\bar{q}Z^{3}\\ +\frac{2(2S_{13}+S_{44})}{{\bar{\lambda }}_{33}}\bar{q}Z^{3}-\frac{3}{10{\left({\bar{\lambda }}_{33}\right)}^{2}}\bar{q}Z-\frac{3(2S_{13}+S_{44})}{10{\bar{\lambda }}_{33}}\bar{q}Z]+{\bar{d}}_{31}{\bar{d}}_{33}(-\frac{4}{{\bar{\lambda }}_{33}}\bar{q}Z^{3}+\frac{3}{5{\bar{\lambda }}_{33}}\bar{q}Z)\\ +{\bar{d}}_{31}{\bar{d}}_{15}(\frac{4}{{\bar{\lambda }}_{33}}\bar{q}Z^{3}-\frac{3}{5{\bar{\lambda }}_{33}}\bar{q}Z)-2S_{13}\bar{q}Z^{3}+\frac{3}{2}S_{13}\bar{q}Z-S_{13}\frac{\bar{q}}{2}-{\bar{d}}_{31}{\bar{d}}_{31}[-\frac{6}{{\bar{\lambda }}_{33}}\bar{q}X^{2}Z\\ -\frac{12}{\bar{b}{\bar{\lambda }}_{33}}\bar{P}XZ+\frac{2}{{\left({\bar{\lambda }}_{33}\right)}^{2}}\bar{q}Z^{3}-\frac{1}{2{\left({\bar{\lambda }}_{33}\right)}^{2}}\bar{q}Z+\frac{2(2S_{13}+S_{44})}{{\bar{\lambda }}_{33}}\bar{q}Z^{3}+\frac{12}{\bar{b}{\bar{\lambda }}_{33}}\bar{M}Z-\frac{3(2S_{13}+S_{44})}{10{\bar{\lambda }}_{33}}\bar{q}Z]\\ -{\bar{d}}_{31}{\bar{d}}_{33}[-\frac{2}{{\bar{\lambda }}_{33}}\bar{q}Z^{3}+\frac{3}{2{\bar{\lambda }}_{33}}\bar{q}Z-\frac{1}{2{\bar{\lambda }}_{33}}\bar{q}]-{\bar{d}}_{31}{\bar{d}}_{15}[\frac{1}{{\bar{\lambda }}_{33}}\bar{q}Z+\frac{2}{{\bar{\lambda }}_{33}}\bar{q}Z^{3}-\frac{3}{2{\bar{\lambda }}_{33}}\bar{q}Z] \end{array}.$$

Integrating Equations (A32) and (A33) respectively, we may obtain

(A34) $$\begin{array}{l} \bar{w}=-\frac{6}{\bar{b}}S_{13}\bar{P}XZ^{2}+\frac{\left(2S_{13}+S_{44}\right)}{2}S_{13}\bar{q}Z^{4}+\frac{6}{\bar{b}}S_{13}\bar{M}Z^{2}-\frac{3(2S_{13}+S_{44})}{20}S_{13}\bar{q}Z^{2}-3S_{13}\bar{q}X^{2}Z^{2}\\ +S_{13}{\left({\bar{d}}_{31}\right)}^{2}[\frac{1}{2{\left({\bar{\lambda }}_{33}\right)}^{2}}\bar{q}Z^{4}+\frac{\left(2S_{13}+S_{44}\right)}{2{\bar{\lambda }}_{33}}\bar{q}Z^{4}-\frac{3}{20{\left({\bar{\lambda }}_{33}\right)}^{2}}\bar{q}Z^{2}-\frac{3(2S_{13}+S_{44})}{20{\bar{\lambda }}_{33}}\bar{q}Z^{2}]\\ +S_{13}{\bar{d}}_{31}{\bar{d}}_{33}(-\frac{1}{{\bar{\lambda }}_{33}}\bar{q}Z^{4}+\frac{3}{10{\bar{\lambda }}_{33}}\bar{q}Z^{2})+S_{13}{\bar{d}}_{31}{\bar{d}}_{15}(\frac{1}{{\bar{\lambda }}_{33}}\bar{q}Z^{4}-\frac{3}{10{\bar{\lambda }}_{33}}\bar{q}Z^{2})-\frac{S_{33}}{2}\bar{q}Z^{4}\\ +\frac{3}{4}S_{33}\bar{q}Z^{2}-S_{33}\frac{\bar{q}}{2}Z-{\bar{d}}_{33}{\bar{d}}_{31}[-\frac{3}{{\bar{\lambda }}_{33}}\bar{q}X^{2}Z^{2}-\frac{6}{\bar{b}{\bar{\lambda }}_{33}}\bar{P}XZ^{2}+\frac{1}{2{\left({\bar{\lambda }}_{33}\right)}^{2}}\bar{q}Z^{4}-\frac{1}{4{\left({\bar{\lambda }}_{33}\right)}^{2}}\bar{q}Z^{2}\\ +\frac{\left(2S_{13}+S_{44}\right)}{2{\bar{\lambda }}_{33}}\bar{q}Z^{4}+\frac{6}{\bar{b}{\bar{\lambda }}_{33}}\bar{M}Z^{2}-\frac{3(2S_{13}+S_{44})}{20{\bar{\lambda }}_{33}}\bar{q}Z^{2}]-{\bar{d}}_{33}{\bar{d}}_{33}[-\frac{1}{2{\bar{\lambda }}_{33}}\bar{q}Z^{4}\\ +\frac{3}{4{\bar{\lambda }}_{33}}\bar{q}Z^{2}-\frac{1}{2{\bar{\lambda }}_{33}}\bar{q}Z]-{\bar{d}}_{33}{\bar{d}}_{15}[\frac{1}{2{\bar{\lambda }}_{33}}\bar{q}Z^{2}+\frac{1}{2{\bar{\lambda }}_{33}}\bar{q}Z^{4}-\frac{3}{4{\bar{\lambda }}_{33}}\bar{q}Z^{2}]+G_{0}(X) \end{array}$$

and

(A35) $$\begin{array}{l} \bar{u}=-2\bar{q}X^{3}Z-\frac{6}{\bar{b}}\bar{P}X^{2}Z+2(2S_{13}+S_{44})\bar{q}XZ^{3}+\frac{12}{\bar{b}}\bar{M}XZ-\frac{3(2S_{13}+S_{44})}{10}\bar{q}XZ\\ +{\left({\bar{d}}_{31}\right)}^{2}[\frac{2}{{\left({\bar{\lambda }}_{33}\right)}^{2}}\bar{q}XZ^{3}+\frac{2(2S_{13}+S_{44})}{{\bar{\lambda }}_{33}}\bar{q}XZ^{3}-\frac{3}{10{\left({\bar{\lambda }}_{33}\right)}^{2}}\bar{q}XZ-\frac{3(2S_{13}+S_{44})}{10{\bar{\lambda }}_{33}}\bar{q}XZ]\\ +{\bar{d}}_{31}{\bar{d}}_{33}(-\frac{4}{{\bar{\lambda }}_{33}}\bar{q}XZ^{3}+\frac{3}{5{\bar{\lambda }}_{33}}\bar{q}XZ)+{\bar{d}}_{31}{\bar{d}}_{15}(\frac{4}{{\bar{\lambda }}_{33}}\bar{q}XZ^{3}-\frac{3}{5{\bar{\lambda }}_{33}}\bar{q}XZ)-2S_{13}\bar{q}XZ^{3}\\ +\frac{3}{2}S_{13}\bar{q}XZ-S_{13}\frac{\bar{q}}{2}X-{\bar{d}}_{31}{\bar{d}}_{31}[-\frac{2}{{\bar{\lambda }}_{33}}\bar{q}X^{3}Z-\frac{6}{\bar{b}{\bar{\lambda }}_{33}}\bar{P}X^{2}Z+\frac{2}{{\left({\bar{\lambda }}_{33}\right)}^{2}}\bar{q}XZ^{3}-\frac{1}{2{\left({\bar{\lambda }}_{33}\right)}^{2}}\bar{q}XZ\\ +\frac{2(2S_{13}+S_{44})}{{\bar{\lambda }}_{33}}\bar{q}XZ^{3}+\frac{12}{\bar{b}{\bar{\lambda }}_{33}}\bar{M}XZ-\frac{3(2S_{13}+S_{44})}{10{\bar{\lambda }}_{33}}\bar{q}XZ]-{\bar{d}}_{31}{\bar{d}}_{33}[-\frac{2}{{\bar{\lambda }}_{33}}\bar{q}XZ^{3}\\ +\frac{3}{2{\bar{\lambda }}_{33}}\bar{q}XZ-\frac{1}{2{\bar{\lambda }}_{33}}\bar{q}X]-{\bar{d}}_{31}{\bar{d}}_{15}[\frac{1}{{\bar{\lambda }}_{33}}\bar{q}XZ+\frac{2}{{\bar{\lambda }}_{33}}\bar{q}XZ^{3}-\frac{3}{2{\bar{\lambda }}_{33}}\bar{q}XZ]+G_{1}(Z) \end{array},$$

From Equations (5) and (16), one has

(A36) $$\begin{array}{l} -2\bar{q}X^{3}+\frac{2}{{\bar{\lambda }}_{33}}{\bar{d}}_{31}{\bar{d}}_{31}\bar{q}X^{3}-\frac{6}{\bar{b}}\bar{P}X^{2}+\frac{6}{\bar{b}{\bar{\lambda }}_{33}}{\bar{d}}_{31}{\bar{d}}_{31}\bar{P}X^{2}+\frac{12}{\bar{b}}\bar{M}X+\frac{9}{10}S_{13}\bar{q}X+\frac{12}{10}S_{44}\bar{q}X\\ +\frac{1}{5{\left({\bar{\lambda }}_{33}\right)}^{2}}{\bar{d}}_{31}{\bar{d}}_{31}\bar{q}X-\frac{9}{10{\bar{\lambda }}_{33}}{\bar{d}}_{31}{\bar{d}}_{33}\bar{q}X+\frac{2}{5{\bar{\lambda }}_{33}}{\bar{d}}_{31}{\bar{d}}_{15}\bar{q}X-\frac{12}{\bar{b}{\bar{\lambda }}_{33}}{\bar{d}}_{31}{\bar{d}}_{31}\bar{M}X-{\bar{d}}_{15}{\bar{d}}_{15}\bar{q}X+\frac{dG_{0}(X)}{dX}\\ =[\frac{6}{\bar{b}}S_{13}\bar{P}+\frac{6}{\bar{b}}S_{44}\bar{P}-\frac{6}{\bar{b}{\bar{\lambda }}_{33}}{\bar{d}}_{33}{\bar{d}}_{31}\bar{P}+\frac{6}{\bar{b}{\bar{\lambda }}_{33}}{\bar{d}}_{15}{\bar{d}}_{31}\bar{P}]Z^{2}-\frac{3}{2\bar{b}}S_{44}\bar{P}-\frac{3}{2\bar{b}{\bar{\lambda }}_{33}}{\bar{d}}_{15}{\bar{d}}_{31}\bar{P}-\frac{dG_{1}(Z)}{dZ}=A \end{array},$$

where *A* is an undetermined constant. From Equation (A36), we have

(A37) $$\left\{ \begin{array}{l} \frac{dG_{0}(X)}{dX}=[2\bar{q}-\frac{2}{{\bar{\lambda }}_{33}}{\bar{d}}_{31}{\bar{d}}_{31}\bar{q}]X^{3}+[\frac{6}{\bar{b}}\bar{P}-\frac{6}{\bar{b}{\bar{\lambda }}_{33}}{\bar{d}}_{31}{\bar{d}}_{31}\bar{P}]X^{2}+[-\frac{12}{\bar{b}}\bar{M}-\frac{9}{10}S_{13}\bar{q}-\frac{12}{10}S_{44}\bar{q}\\ -\frac{1}{5{\left({\bar{\lambda }}_{33}\right)}^{2}}{\bar{d}}_{31}{\bar{d}}_{31}\bar{q}+\frac{9}{10{\bar{\lambda }}_{33}}{\bar{d}}_{31}{\bar{d}}_{33}\bar{q}-\frac{2}{5{\bar{\lambda }}_{33}}{\bar{d}}_{31}{\bar{d}}_{15}\bar{q}+\frac{12}{\bar{b}{\bar{\lambda }}_{33}}{\bar{d}}_{31}{\bar{d}}_{31}\bar{M}+{\bar{d}}_{15}{\bar{d}}_{15}\bar{q}]X+A\\ \frac{dG_{1}(Z)}{dZ}=[\frac{6}{\bar{b}}S_{13}\bar{P}+\frac{6}{\bar{b}}S_{44}\bar{P}-\frac{6}{\bar{b}{\bar{\lambda }}_{33}}{\bar{d}}_{33}{\bar{d}}_{31}\bar{P}+\frac{6}{\bar{b}{\bar{\lambda }}_{33}}{\bar{d}}_{15}{\bar{d}}_{31}\bar{P}]Z^{2}-\frac{3}{2\bar{b}}S_{44}\bar{P}-\frac{3}{2\bar{b}{\bar{\lambda }}_{33}}{\bar{d}}_{15}{\bar{d}}_{31}\bar{P}-A \end{array}\right..$$

Integrating Equation (A37), it can be obtained

(A38) $$\left\{ \begin{array}{l} G_{0}(X)=[\frac{1}{2}\bar{q}-\frac{1}{2{\bar{\lambda }}_{33}}{\bar{d}}_{31}{\bar{d}}_{31}\bar{q}]X^{4}+[\frac{2}{\bar{b}}\bar{P}-\frac{2}{\bar{b}{\bar{\lambda }}_{33}}{\bar{d}}_{31}{\bar{d}}_{31}\bar{P}]X^{3}+[-\frac{6}{\bar{b}}\bar{M}-\frac{9}{20}S_{13}\bar{q}-\frac{6}{10}S_{44}\bar{q}\\ -\frac{1}{10{\left({\bar{\lambda }}_{33}\right)}^{2}}{\bar{d}}_{31}{\bar{d}}_{31}\bar{q}+\frac{9}{20{\bar{\lambda }}_{33}}{\bar{d}}_{31}{\bar{d}}_{33}\bar{q}-\frac{1}{5{\bar{\lambda }}_{33}}{\bar{d}}_{31}{\bar{d}}_{15}\bar{q}+\frac{6}{\bar{b}{\bar{\lambda }}_{33}}{\bar{d}}_{31}{\bar{d}}_{31}\bar{M}+\frac{1}{2}{\bar{d}}_{15}{\bar{d}}_{15}\bar{q}]X^{2}+AX+B\\ G_{1}(Z)=[\frac{2}{\bar{b}}S_{13}\bar{P}+\frac{2}{\bar{b}}S_{44}\bar{P}-\frac{2}{\bar{b}{\bar{\lambda }}_{33}}{\bar{d}}_{33}{\bar{d}}_{31}\bar{P}+\frac{2}{\bar{b}{\bar{\lambda }}_{33}}{\bar{d}}_{15}{\bar{d}}_{31}\bar{P}]Z^{3}\\ +[-\frac{3}{2\bar{b}}S_{44}\bar{P}-\frac{3}{2\bar{b}{\bar{\lambda }}_{33}}{\bar{d}}_{15}{\bar{d}}_{31}\bar{P}-A]Z+C \end{array}\right..$$

Thus, we can finally obtain

(A39) $$\begin{array}{l} \bar{w}=-3S_{13}\bar{q}X^{2}Z^{2}-\frac{6}{\bar{b}}S_{13}\bar{P}XZ^{2}+\frac{\left(2S_{13}+S_{44}\right)}{2}S_{13}\bar{q}Z^{4}+\frac{6}{\bar{b}}S_{13}\bar{M}Z^{2}-\frac{3(2S_{13}+S_{44})}{20}S_{13}\bar{q}Z^{2}\\ +S_{13}{\left({\bar{d}}_{31}\right)}^{2}[\frac{1}{2{\left({\bar{\lambda }}_{33}\right)}^{2}}\bar{q}Z^{4}+\frac{\left(2S_{13}+S_{44}\right)}{2{\bar{\lambda }}_{33}}\bar{q}Z^{4}-\frac{3}{20{\left({\bar{\lambda }}_{33}\right)}^{2}}\bar{q}Z^{2}-\frac{3(2S_{13}+S_{44})}{20{\bar{\lambda }}_{33}}\bar{q}Z^{2}]-\frac{S_{33}}{2}\bar{q}Z^{4}\\ +S_{13}{\bar{d}}_{31}{\bar{d}}_{33}(-\frac{1}{{\bar{\lambda }}_{33}}\bar{q}Z^{4}+\frac{3}{10{\bar{\lambda }}_{33}}\bar{q}Z^{2})+S_{13}{\bar{d}}_{31}{\bar{d}}_{15}(\frac{1}{{\bar{\lambda }}_{33}}\bar{q}Z^{4}-\frac{3}{10{\bar{\lambda }}_{33}}\bar{q}Z^{2})+\frac{3}{4}S_{33}\bar{q}Z^{2}-S_{33}\frac{\bar{q}}{2}Z\\ -{\bar{d}}_{33}{\bar{d}}_{31}[-\frac{3}{{\bar{\lambda }}_{33}}\bar{q}X^{2}Z^{2}-\frac{6}{\bar{b}{\bar{\lambda }}_{33}}\bar{P}XZ^{2}+\frac{1}{2{\left({\bar{\lambda }}_{33}\right)}^{2}}\bar{q}Z^{4}-\frac{1}{4{\left({\bar{\lambda }}_{33}\right)}^{2}}\bar{q}Z^{2}+\frac{\left(2S_{13}+S_{44}\right)}{2{\bar{\lambda }}_{33}}\bar{q}Z^{4}+\frac{6}{\bar{b}{\bar{\lambda }}_{33}}\bar{M}Z^{2}\\ -\frac{3(2S_{13}+S_{44})}{20{\bar{\lambda }}_{33}}\bar{q}Z^{2}]-{\bar{d}}_{33}{\bar{d}}_{33}[-\frac{1}{2{\bar{\lambda }}_{33}}\bar{q}Z^{4}+\frac{3}{4{\bar{\lambda }}_{33}}\bar{q}Z^{2}-\frac{1}{2{\bar{\lambda }}_{33}}\bar{q}Z]-{\bar{d}}_{33}{\bar{d}}_{15}[\frac{1}{2{\bar{\lambda }}_{33}}\bar{q}Z^{2}+\frac{1}{2{\bar{\lambda }}_{33}}\bar{q}Z^{4}\\ -\frac{3}{4{\bar{\lambda }}_{33}}\bar{q}Z^{2}]+[\frac{1}{2}\bar{q}-\frac{1}{2{\bar{\lambda }}_{33}}{\bar{d}}_{31}{\bar{d}}_{31}\bar{q}]X^{4}+[\frac{2}{\bar{b}}\bar{P}-\frac{2}{\bar{b}{\bar{\lambda }}_{33}}{\bar{d}}_{31}{\bar{d}}_{31}\bar{P}]X^{3}+[-\frac{6}{\bar{b}}\bar{M}-\frac{9}{20}S_{13}\bar{q}-\frac{6}{10}S_{44}\bar{q}\\ -\frac{1}{10{\left({\bar{\lambda }}_{33}\right)}^{2}}{\bar{d}}_{31}{\bar{d}}_{31}\bar{q}+\frac{9}{20{\bar{\lambda }}_{33}}{\bar{d}}_{31}{\bar{d}}_{33}\bar{q}-\frac{1}{5{\bar{\lambda }}_{33}}{\bar{d}}_{31}{\bar{d}}_{15}\bar{q}+\frac{6}{\bar{b}{\bar{\lambda }}_{33}}{\bar{d}}_{31}{\bar{d}}_{31}\bar{M}+\frac{1}{2}{\bar{d}}_{15}{\bar{d}}_{15}\bar{q}]X^{2}+AX+B \end{array}$$

and

(A40) $$\begin{array}{l} \bar{u}=-2\bar{q}X^{3}Z-\frac{6}{\bar{b}}\bar{P}X^{2}Z+2(2S_{13}+S_{44})\bar{q}XZ^{3}+\frac{12}{\bar{b}}\bar{M}XZ-\frac{3(2S_{13}+S_{44})}{10}\bar{q}XZ-2S_{13}\bar{q}XZ^{3}\\ +{\left({\bar{d}}_{31}\right)}^{2}[\frac{2}{{\left({\bar{\lambda }}_{33}\right)}^{2}}\bar{q}XZ^{3}+\frac{2(2S_{13}+S_{44})}{{\bar{\lambda }}_{33}}\bar{q}XZ^{3}-\frac{3}{10{\left({\bar{\lambda }}_{33}\right)}^{2}}\bar{q}XZ-\frac{3(2S_{13}+S_{44})}{10{\bar{\lambda }}_{33}}\bar{q}XZ]-S_{13}\frac{\bar{q}}{2}X\\ +{\bar{d}}_{31}{\bar{d}}_{33}(-\frac{4}{{\bar{\lambda }}_{33}}\bar{q}XZ^{3}+\frac{3}{5{\bar{\lambda }}_{33}}\bar{q}XZ)+{\bar{d}}_{31}{\bar{d}}_{15}(\frac{4}{{\bar{\lambda }}_{33}}\bar{q}XZ^{3}-\frac{3}{5{\bar{\lambda }}_{33}}\bar{q}XZ)+\frac{3}{2}S_{13}\bar{q}XZ\\ -{\bar{d}}_{31}{\bar{d}}_{31}[-\frac{2}{{\bar{\lambda }}_{33}}\bar{q}X^{3}Z-\frac{6}{\bar{b}{\bar{\lambda }}_{33}}\bar{P}X^{2}Z+\frac{2}{{\left({\bar{\lambda }}_{33}\right)}^{2}}\bar{q}XZ^{3}-\frac{1}{2{\left({\bar{\lambda }}_{33}\right)}^{2}}\bar{q}XZ+\frac{2(2S_{13}+S_{44})}{{\bar{\lambda }}_{33}}\bar{q}XZ^{3}\\ +\frac{12}{\bar{b}{\bar{\lambda }}_{33}}\bar{M}XZ-\frac{3(2S_{13}+S_{44})}{10{\bar{\lambda }}_{33}}\bar{q}XZ]-{\bar{d}}_{31}{\bar{d}}_{33}[-\frac{2}{{\bar{\lambda }}_{33}}\bar{q}XZ^{3}+\frac{3}{2{\bar{\lambda }}_{33}}\bar{q}XZ-\frac{1}{2{\bar{\lambda }}_{33}}\bar{q}X]\\ -{\bar{d}}_{31}{\bar{d}}_{15}[\frac{1}{{\bar{\lambda }}_{33}}\bar{q}XZ+\frac{2}{{\bar{\lambda }}_{33}}\bar{q}XZ^{3}-\frac{3}{2{\bar{\lambda }}_{33}}\bar{q}XZ]+[\frac{2}{\bar{b}}S_{13}\bar{P}+\frac{2}{\bar{b}}S_{44}\bar{P}-\frac{2}{\bar{b}{\bar{\lambda }}_{33}}{\bar{d}}_{33}{\bar{d}}_{31}\bar{P}\\ +\frac{2}{\bar{b}{\bar{\lambda }}_{33}}{\bar{d}}_{15}{\bar{d}}_{31}\bar{P}]Z^{3}+[-\frac{3}{2\bar{b}}S_{44}\bar{P}-\frac{3}{2\bar{b}{\bar{\lambda }}_{33}}{\bar{d}}_{15}{\bar{d}}_{31}\bar{P}-A]Z+C \end{array}.$$

Substituting Equations (A39) and (A40) into Equation (24), we have

(A41) $$\begin{array}{l} A=-[2\bar{q}-\frac{2}{{\bar{\lambda }}_{33}}{\bar{d}}_{31}{\bar{d}}_{31}\bar{q}]\frac{l^{3}}{h^{3}}-[\frac{6}{\bar{b}}\bar{P}-\frac{6}{\bar{b}{\bar{\lambda }}_{33}}{\bar{d}}_{31}{\bar{d}}_{31}\bar{P}]\frac{l^{2}}{h^{2}}-[-\frac{12}{\bar{b}}\bar{M}-\frac{9}{10}S_{13}\bar{q}-\frac{6}{5}S_{44}\bar{q}\\ -\frac{1}{5{\left({\bar{\lambda }}_{33}\right)}^{2}}{\bar{d}}_{31}{\bar{d}}_{31}\bar{q}+\frac{9}{10{\bar{\lambda }}_{33}}{\bar{d}}_{31}{\bar{d}}_{33}\bar{q}-\frac{2}{5{\bar{\lambda }}_{33}}{\bar{d}}_{31}{\bar{d}}_{15}\bar{q}+\frac{12}{\bar{b}{\bar{\lambda }}_{33}}{\bar{d}}_{31}{\bar{d}}_{31}\bar{M}+{\bar{d}}_{15}{\bar{d}}_{15}\bar{q}]\frac{l}{h} \end{array},$$

(A42) $$\begin{array}{l} B=[\frac{3}{2}\bar{q}-\frac{3}{2{\bar{\lambda }}_{33}}{\bar{d}}_{31}{\bar{d}}_{31}\bar{q}]\frac{l^{4}}{h^{4}}+[\frac{4}{\bar{b}}\bar{P}-\frac{4}{\bar{b}{\bar{\lambda }}_{33}}{\bar{d}}_{31}{\bar{d}}_{31}\bar{P}]\frac{l^{3}}{h^{3}}-[\frac{6}{\bar{b}}\bar{M}+\frac{9}{20}S_{13}\bar{q}+\frac{6}{10}S_{44}\bar{q}\\ +\frac{1}{10{\left({\bar{\lambda }}_{33}\right)}^{2}}{\bar{d}}_{31}{\bar{d}}_{31}\bar{q}-\frac{9}{20{\bar{\lambda }}_{33}}{\bar{d}}_{31}{\bar{d}}_{33}\bar{q}+\frac{1}{5{\bar{\lambda }}_{33}}{\bar{d}}_{31}{\bar{d}}_{15}\bar{q}-\frac{6}{\bar{b}{\bar{\lambda }}_{33}}{\bar{d}}_{31}{\bar{d}}_{31}\bar{M}-\frac{1}{2}{\bar{d}}_{15}{\bar{d}}_{15}\bar{q}]\frac{l^{2}}{h^{2}} \end{array},$$

and

(A43) $$C=S_{13}\frac{\bar{q}}{2}\frac{l}{h}-\frac{1}{2{\bar{\lambda }}_{33}}{\bar{d}}_{31}{\bar{d}}_{33}\bar{q}\frac{l}{h}.$$

From Equations (16), (A41), (A42), and (A43), Equations (A39) and (A40) can be transformed into

(A44) $$\begin{array}{l} w=-3s_{13}q\frac{1}{h^{3}}x^{2}z^{2}-\frac{6}{b}s_{13}P\frac{1}{h^{3}}xz^{2}+\frac{\left(2s_{13}+s_{44}\right)}{2s_{11}}s_{13}q\frac{1}{h^{3}}z^{4}+\frac{6}{b}s_{13}\frac{M}{h^{3}}z^{2}-\frac{3(2s_{13}+s_{44})}{20s_{11}}s_{13}q\frac{1}{h}z^{2}\\ +\frac{\lambda_{11}}{2{\left(\lambda_{33}\right)}^{2}}\frac{s_{13}}{s_{11}}{\left(d_{31}\right)}^{2}q\frac{1}{h^{3}}z^{4}+\frac{(2s_{13}+s_{44})s_{13}}{2\lambda_{33}{\left(s_{11}\right)}^{2}}q{\left(d_{31}\right)}^{2}\frac{1}{h^{3}}z^{4}-\frac{3\lambda_{11}s_{13}}{20{\left(\lambda_{33}\right)}^{2}s_{11}}{\left(d_{31}\right)}^{2}q\frac{1}{h}z^{2}-\frac{s_{33}}{2}qz\\ -\frac{3(2s_{13}+s_{44})}{20\lambda_{33}{\left(s_{11}\right)}^{2}}s_{13}{\left(d_{31}\right)}^{2}q\frac{1}{h}z^{2}-\frac{s_{13}}{\lambda_{33}s_{11}}d_{31}d_{33}q\frac{1}{h^{3}}z^{4}+\frac{3s_{13}}{10\lambda_{33}s_{11}}d_{31}d_{33}q\frac{1}{h}z^{2}-\frac{s_{33}}{2}q\frac{1}{h^{3}}z^{4}\\ +\frac{s_{13}}{\lambda_{33}s_{11}}d_{31}d_{15}q\frac{1}{h^{3}}z^{4}-\frac{3s_{13}}{10\lambda_{33}s_{11}}d_{31}d_{15}q\frac{1}{h}z^{2}+\frac{3s_{33}}{4}q\frac{1}{h}z^{2}+\frac{3}{\lambda_{33}}d_{33}d_{31}q\frac{1}{h^{3}}x^{2}z^{2}\\ +\frac{6}{b\lambda_{33}}d_{33}d_{31}\frac{P}{h^{3}}xz^{2}-\frac{\lambda_{11}}{2{\left(\lambda_{33}\right)}^{2}}d_{33}d_{31}q\frac{1}{h^{3}}z^{4}+\frac{\lambda_{11}}{4{\left(\lambda_{33}\right)}^{2}}d_{33}d_{31}q\frac{1}{h}z^{2}-\frac{6}{b\lambda_{33}}d_{33}d_{31}\frac{M}{h^{3}}z^{2}\\ -\frac{\left(2s_{13}+s_{44}\right)}{2\lambda_{33}s_{11}}d_{33}d_{31}q\frac{1}{h^{3}}z^{4}+\frac{3(2s_{13}+s_{44})}{20\lambda_{33}s_{11}}d_{33}d_{31}q\frac{1}{h}z^{2}+\frac{1}{2\lambda_{33}}d_{33}d_{33}q\frac{1}{h^{3}}z^{4}\\ -\frac{3}{4\lambda_{33}}d_{33}d_{33}q\frac{1}{h}z^{2}+\frac{1}{2\lambda_{33}}d_{33}d_{33}qz-\frac{1}{2\lambda_{33}}d_{33}d_{15}q\frac{1}{h}z^{2}-\frac{1}{2\lambda_{33}}d_{33}d_{15}q\frac{1}{h^{3}}z^{4}\\ +\frac{3}{4\lambda_{33}}d_{33}d_{15}q\frac{1}{h}z^{2}+[\frac{1}{2}qs_{11}-\frac{1}{2\lambda_{33}}d_{31}d_{31}q]\frac{1}{h^{3}}x^{4}+[\frac{2}{b}\frac{P}{h}s_{11}-\frac{2}{b\lambda_{33}}d_{31}d_{31}\frac{P}{h}]\frac{1}{h^{2}}x^{3}\\ +[-\frac{6}{b}\frac{M}{h^{2}}s_{11}-\frac{9}{20}s_{13}q-\frac{6}{10}s_{44}q-\frac{\lambda_{11}}{10{\left(\lambda_{33}\right)}^{2}}d_{31}d_{31}q+\frac{9}{20\lambda_{33}}d_{31}d_{33}q-\frac{1}{5\lambda_{33}}d_{31}d_{15}q\\ +\frac{6}{b\lambda_{33}}d_{31}d_{31}\frac{M}{h^{2}}+\frac{1}{2\lambda_{11}}d_{15}d_{15}q]\frac{1}{h}x^{2}-\{[2qs_{11}-\frac{2}{\lambda_{33}}d_{31}d_{31}q]\frac{l^{3}}{h^{3}}+[\frac{6}{b}\frac{P}{h}s_{11}\\ -\frac{6}{b\lambda_{33}}d_{31}d_{31}\frac{P}{h}]\frac{l^{2}}{h^{2}}+[-\frac{12}{b}\frac{M}{h^{2}}s_{11}-\frac{9}{10}s_{13}q-\frac{6}{5}s_{44}q-\frac{\lambda_{11}}{5{\left(\lambda_{33}\right)}^{2}}d_{31}d_{31}q+\frac{9}{10\lambda_{33}}d_{31}d_{33}q\\ -\frac{2}{5\lambda_{33}}d_{31}d_{15}q+\frac{12}{b\lambda_{33}}d_{31}d_{31}\frac{M}{h^{2}}+\frac{1}{\lambda_{11}}d_{15}d_{15}q]\frac{l}{h}\}x+[\frac{3}{2}qs_{11}-\frac{3}{2\lambda_{33}}d_{31}d_{31}q]\frac{l^{4}}{h^{3}}\\ +[\frac{4}{b}\frac{P}{h}s_{11}-\frac{4}{b\lambda_{33}}d_{31}d_{31}\frac{P}{h}]\frac{l^{3}}{h^{2}}-[\frac{6}{b}\frac{M}{h^{2}}s_{11}+\frac{9}{20}s_{13}q+\frac{6}{10}s_{44}q+\frac{\lambda_{11}}{10{\left(\lambda_{33}\right)}^{2}}d_{31}d_{31}q\\ -\frac{9}{20\lambda_{33}}d_{31}d_{33}q+\frac{1}{5\lambda_{33}}d_{31}d_{15}q-\frac{6}{b\lambda_{33}}d_{31}d_{31}\frac{M}{h^{2}}-\frac{1}{2\lambda_{11}}d_{15}d_{15}q]\frac{l^{2}}{h} \end{array}$$

and

(A45) $$\begin{array}{l} u=-2qs_{11}\frac{1}{h^{3}}x^{3}z-\frac{6}{b}\frac{P}{h^{3}}s_{11}x^{2}z+2(2s_{13}+s_{44})q\frac{1}{h^{3}}xz^{3}+\frac{12}{b}\frac{M}{h^{3}}s_{11}xz+\frac{3}{2}s_{13}q\frac{1}{h}xz\\ -\frac{3(2s_{13}+s_{44})}{10}q\frac{1}{h}xz+\frac{2\lambda_{11}}{{\left(\lambda_{33}\right)}^{2}}{\left(d_{31}\right)}^{2}q\frac{1}{h^{3}}xz^{3}+\frac{2(2s_{13}+s_{44})}{\lambda_{33}s_{11}}{\left(d_{31}\right)}^{2}q\frac{1}{h^{3}}xz^{3}-s_{13}\frac{q}{2}x\\ -\frac{3\lambda_{11}}{10{\left(\lambda_{33}\right)}^{2}}{\left(d_{31}\right)}^{2}q\frac{1}{h}xz-\frac{3(2s_{13}+s_{44})}{10\lambda_{33}s_{11}}{\left(d_{31}\right)}^{2}q\frac{1}{h}xz-\frac{4}{\lambda_{33}}d_{31}d_{33}q\frac{1}{h^{3}}xz^{3}-\frac{1}{2\lambda_{33}}d_{31}d_{33}ql\\ +\frac{3}{5\lambda_{33}}d_{31}d_{33}q\frac{1}{h}xz+\frac{4}{\lambda_{33}}d_{31}d_{15}q\frac{1}{h^{3}}xz^{3}-\frac{3}{5\lambda_{33}}d_{31}d_{15}q\frac{1}{h}xz-2s_{13}q\frac{1}{h^{3}}xz^{3}\\ +\frac{2}{\lambda_{33}}d_{31}d_{31}q\frac{1}{h^{3}}x^{3}z+\frac{6}{b\lambda_{33}}d_{31}d_{31}\frac{P}{h^{3}}x^{2}z-\frac{2\lambda_{11}}{{\left(\lambda_{33}\right)}^{2}}d_{31}d_{31}q\frac{1}{h^{3}}xz^{3}+\frac{1}{2\lambda_{33}}d_{31}d_{33}qx\\ +\frac{\lambda_{11}}{2{\left(\lambda_{33}\right)}^{2}}d_{31}d_{31}q\frac{1}{h}xz-\frac{2(2s_{13}+s_{44})}{\lambda_{33}s_{11}}d_{31}d_{31}q\frac{1}{h^{3}}xz^{3}-\frac{12}{b\lambda_{33}}d_{31}d_{31}\frac{M}{h^{3}}xz\\ +\frac{3(2s_{13}+s_{44})}{10\lambda_{33}s_{11}}d_{31}d_{31}q\frac{1}{h}xz+\frac{2}{\lambda_{33}}d_{31}d_{33}q\frac{1}{h^{3}}xz^{3}-\frac{3}{2\lambda_{33}}d_{31}d_{33}q\frac{1}{h}xz+s_{13}\frac{q}{2}l\\ -\frac{1}{\lambda_{33}}d_{31}d_{15}q\frac{1}{h}xz-\frac{2}{\lambda_{33}}d_{31}d_{15}q\frac{1}{h^{3}}xz^{3}+\frac{3}{2\lambda_{33}}d_{31}d_{15}q\frac{1}{h}xz+[\frac{2}{b}s_{13}\frac{P}{h}+\frac{2}{b}s_{44}\frac{P}{h}\\ -\frac{2}{b\lambda_{33}}d_{33}d_{31}\frac{P}{h}+\frac{2}{b\lambda_{33}}d_{15}d_{31}\frac{P}{h}]\frac{1}{h^{2}}z^{3}+\{-\frac{3}{2b}s_{44}\frac{P}{h}-\frac{3}{2b\lambda_{33}}d_{15}d_{31}\frac{P}{h}+[2qs_{11}\\ -\frac{2}{\lambda_{33}}d_{31}d_{31}q]\frac{l^{3}}{h^{3}}+[\frac{6}{b}\frac{P}{h}s_{11}-\frac{6}{b\lambda_{33}}d_{31}d_{31}\frac{P}{h}]\frac{l^{2}}{h^{2}}+[-\frac{12}{b}\frac{M}{h^{2}}s_{11}-\frac{9}{10}s_{13}q-\frac{6}{5}s_{44}q\\ -\frac{\lambda_{11}}{5{\left(\lambda_{33}\right)}^{2}}d_{31}d_{31}q+\frac{9}{10\lambda_{33}}d_{31}d_{33}q-\frac{2}{5\lambda_{33}}d_{31}d_{15}q+\frac{12}{b\lambda_{33}}d_{31}d_{31}\frac{M}{h^{2}}+\frac{1}{\lambda_{11}}d_{15}d_{15}q]\frac{l}{h}\}z \end{array}.$$

Similarly, the expression of stress components can be obtained

(A46) $$\begin{array}{l} \sigma_{x}=-6q\frac{1}{h^{3}}x^{2}z-\frac{12}{b}\frac{P}{h^{3}}xz+[\frac{2(2s_{13}+s_{44})}{s_{11}}q\frac{1}{h^{3}}+\frac{2\lambda_{11}}{s_{11}{\left(\lambda_{33}\right)}^{2}}{\left(d_{31}\right)}^{2}q\frac{1}{h^{3}}-\frac{4}{\lambda_{33}s_{11}}d_{31}d_{33}q\frac{1}{h^{3}}\\ +\frac{2(2s_{13}+s_{44})}{\lambda_{33}{\left(s_{11}\right)}^{2}}{\left(d_{31}\right)}^{2}q\frac{1}{h^{3}}+\frac{4}{\lambda_{33}s_{11}}d_{31}d_{15}q\frac{1}{h^{3}}]z^{3}+[\frac{12}{b}\frac{M}{h^{3}}-\frac{3(2s_{13}+s_{44})}{10s_{11}}q\frac{1}{h}\\ -\frac{3\lambda_{11}}{10s_{11}{\left(\lambda_{33}\right)}^{2}}{\left(d_{31}\right)}^{2}q\frac{1}{h}-\frac{3(2s_{13}+s_{44})}{10\lambda_{33}{\left(s_{11}\right)}^{2}}{\left(d_{31}\right)}^{2}q\frac{1}{h}+\frac{3}{5\lambda_{33}s_{11}}d_{31}d_{33}q\frac{1}{h}-\frac{3}{5\lambda_{33}s_{11}}d_{31}d_{15}q\frac{1}{h}]z \end{array},$$

(A47) $$\sigma_{z}=-\frac{2q}{h^{3}}z^{3}+\frac{3q}{2h}z-\frac{q}{2},$$

and

(A48) $$\tau_{zx}=\frac{6q}{h^{3}}xz^{2}-\frac{3q}{2h}x+\frac{6P}{bh^{3}}z^{2}-\frac{3P}{2bh}.$$

The expressions of electric displacement components are

(A49) $$D_{x}=(d_{15}+\frac{\lambda_{11}d_{31}}{\lambda_{33}})(\frac{6q}{h^{3}}xz^{2}-\frac{3q}{2h}x+\frac{6P}{bh^{3}}z^{2}-\frac{3P}{2bh})$$

and

(A50) $$\begin{array}{l} D_{z}=[\frac{2\lambda_{11}}{s_{11}{\left(\lambda_{33}\right)}^{2}}{\left(d_{31}\right)}^{3}q\frac{1}{h^{3}}+\frac{2(2s_{13}+s_{44})}{\lambda_{33}{\left(s_{11}\right)}^{2}}{\left(d_{31}\right)}^{3}q\frac{1}{h^{3}}-\frac{4}{\lambda_{33}s_{11}}d_{31}d_{31}d_{33}q\frac{1}{h^{3}}-\frac{2\lambda_{11}d_{31}q}{\lambda_{33}h^{3}}\\ +\frac{4}{\lambda_{33}s_{11}}d_{31}d_{31}d_{15}q\frac{1}{h^{3}}-\frac{2d_{15}q}{h^{3}}]z^{3}+d_{31}[-\frac{3\lambda_{11}}{10s_{11}{\left(\lambda_{33}\right)}^{2}}{\left(d_{31}\right)}^{3}q\frac{1}{h}-\frac{3(2s_{13}+s_{44})}{10\lambda_{33}{\left(s_{11}\right)}^{2}}{\left(d_{31}\right)}^{3}q\frac{1}{h}\\ +\frac{3}{5\lambda_{33}s_{11}}d_{31}d_{31}d_{33}q\frac{1}{h}-\frac{3}{5\lambda_{33}s_{11}}d_{31}d_{31}d_{15}q\frac{1}{h}+\frac{\lambda_{11}d_{31}q}{2\lambda_{33}h}+\frac{d_{15}q}{2h}]z \end{array}.$$

## References

[1] Jiang Y.G., Gong L.L., Hu X.H., Zhao Y., Chen H.W., Feng L., Zhang D.Y. (2018). Aligned P(VDF-TrFE) nanofibers for enhanced piezoelectric directional strain sensors. Polymers.

[2] Elnabawy E., Hassanain A., Shehata N., Popelka A., Nair R., Yousef S., Kandas I. (2019). Piezoelastic PVDF/TPU nanofibrous composite membrane: Fabrication and characterization. Polymers.

[3] Oh W.J., Lim H.S., Won J.S., Lee S.G. (2018). Preparation of PVDF/PAR composites with piezoelectric properties by post-treatment. Polymers.

[4] Kim M., Wu Y.S., Kan E.C., Fan J. (2018). Breathable and flexible piezoelectric ZnO@PVDF fibrous nanogenerator for wearable applications. Polymers.

[5] Moghadam A., Kouzani A., Zamani R., Magniez K., Kaynak A. (2015). Nonlinear large deformation dynamic analysis of electroactive polymer actuators. Smart Struct. Syst..

[6] Nasri-Nasrabadi B., Kaynak A., Komeily-Nia Z., Li J., Zolfagharian A., Adams S., Kouzani A. (2018). An electroactive polymer composite with reinforced bending strength, based on tubular micro carbonized-cellulose. Chem. Eng. J..

[7] Gibeau B., Koch C.R., Ghaemi S. (2019). Active control of vortex shedding from a blunt trailing edge using oscillating piezoelectric flaps. Phys. Rev. Fluids.

[8] Moretti M., Silva E.C.N., Reddy J.N. (2019). Topology optimization of flex tensional piezoelectric actuators with active control law. Smart Mater. Struct..

[9] Ji H.L., Qiu J.H., Wu Y.P., Zhang C. (2019). Semi-active vibration control based on synchronously switched piezoelectric actuators. Int. J. Appl. Electrom..

[10] Wang Z.K., Chen G.C. (1994). A general solution and the application of space axisymmetric problem in piezoelectric material. Appl. Math. Mech..

[11] Lin Q.R., Liu Z.X., Jin Z.L. (2000). A close form solution to simply supported piezoelectric beams under uniform exterior pressure. Appl. Math. Mech..

[12] Mei F.L., Zeng D.S. (2002). State equation method of mechanical-electric coupling for a piezoelectric beam. J. Shandong Univ. Sci. Technol..

[13] Zhu C.Z. (2001). Analytic solution to piezoelectric cantilever beam with concentrated force at free end. J. Nanjing Inst. Technol..

[14] Ding H.J., Jiang A.M. (2005). Polynomial solutions to piezoelectric beams(Ι)-several exact solutions. Appl. Math. Mech..

[15] Ding H.J., Jiang A.M. (2005). Polynomial solutions to piezoelectric beams(ΙΙ)-Analytical solutions to typical problems. Appl. Math. Mech..

[16] Ding H.J., Wang G.Q., Chen W.Q. (1997). Green’s functions for a two-phase infinite piezoelectric plane. Proc. R. Soc..

[17] Yang D.Q., Liu Z.X. (2003). Analytical solution for bending of a piezoelectric cantilever beam under an end load. Chin. Q. Mech..

[18] Pang X.M., Qiu J.H., Zhu K.J., Du J.Z. (2012). (K, Na)NbO_3_-based lead-free piezoelectric ceramics manufactured by two-step sintering. Ceram. Int..

[19] Zhu Q., Yue J.Z., Liu W.Q., Wang X.D., Chen J., Hu G.D. (2017). Active vibration control for piezoelectricity cantilever beam: an adaptive feed forward control method. Smart Mater. Struct..

[20] Peng J., Zhang G., Xiang M.J., Sun H.X., Wang X.Y., Xie X.Z. (2019). Vibration control for the nonlinear resonant response of a piezoelectric elastic beam via time-delayed feedback. Smart Mater. Struct..

[21] Liu Y.J., Yang D.Q. (2002). Analytical solution of the bending problem of piezoelectricity cantilever beam under uniformly distributed loading. Acta Mech. Solida Sin..

[22] Huang B.B., Shi Z.F. (2002). Several analytical solutions for a functionally gradient piezoelectric cantilever. Acta Mater. Compos. Sin..

[23] Zhang L.N., Shi Z.F. (2002). Analytical solution of simply-supported gradient piezoelectric beam. J. North. Jiaotong Univ..

[24] Wang Q., Quek S.T., Sun C.T., Liu X. (2001). Analysis of piezoelectric coupled circular plate. Smart Mater. Struct..

[25] Lian Y.S., He X.T., Shi S.J., Li X., Yang Z.X., Sun J.Y. (2018). A multi-parameter perturbation solution for functionally graded piezoelectric cantilever beams under combined loads. Materials.

[26] Chen S.L., Li Q.Z. (2003). The FPPM solutions for the problems of large deflection of axisymmetric circular plate. J. Chongqing Jianzhu Univ..

[27] Lian Y.S., He X.T., Liu G.H., Sun J.Y., Zheng Z.L. (2017). Application of perturbation idea to well-known Hencky problem: A perturbation solution without small-rotation-angle assumption. Mech. Res. Commun..

[28] Nowinski J.L., Ismail I.A. (1965). Application of a multi-parameter perturbation method to elastostatics. J Theor. App. Mech..

[29] Chien W.Z. (2002). Second order approximation solution of nonlinear large deflection problem of Yongjiang Railway Bridge in Ningbo. Appl. Math. Mech..

[30] He X.T., Chen S.L. (2006). Biparametric perturbation solutions of the large deflection problem of cantilever beams. Appl. Math. Mech..

[31] He X.T., Cao L., Li Z.Y., Hu X.J., Sun J.Y. (2013). Nonlinear large deflection problems of beams with gradient: A biparametric perturbation method. App. Math. Comput..

[32] He X.T., Cao L., Sun J.Y., Zheng Z.L. (2014). Application of a biparametric perturbation method to large-deflection circular plate problems with a bimodular effect under combined loads. J. Math. Anal. Appl..

[33] Ruan X.P., Danforth S.C., Safari A., Chou T.W. (2000). Saint-Venant end effects in piezoceramic materials. Int. J. Solids Struct..

[34] He X.T., Yang Z.X., Jing H.X., Sun J.Y. (2019). One-dimensional theoretical solution and two-dimensional numerical simulation for functionally-graded piezoelectric cantilever beams with different properties in tension and compression. Polymers.

